# The fertility of a sub-population of stallions is negatively affected by ambient climatic conditions, mediated through DNA damage

**DOI:** 10.1007/s00484-025-03123-y

**Published:** 2026-03-09

**Authors:** Róisín A. Griffin, Kasey Miller, Kim Colyvas, Alecia Sheridan, Geoffry De Iuliis, Robert John Aitken, Mark A. Baker, Zamira Gibb, Aleona Swegen

**Affiliations:** 1 Infertility and Reproduction Research Program, Hunter Medical Research Institute, New Lambton Heights, Australia; 2 School of Environment and Life Sciences, College of Engineering, Science and Environment, University of Newcastle, Callaghan, Australia; 3 School of Engineering (Statistics), College of Engineering, Science and Environment, University of Newcastle, Callaghan, Australia; 4 School of Biomedical Sciences and Pharmacy, College of Health, Medicine and Wellbeing, University of Newcastle, Callaghan, Australia

**Keywords:** Male fertility, Male subfertility, Spermatozoa, Heat load, Housing

## Abstract

Exposure to systemic heat stress in male mammals adversely affects sperm production, fertility, and DNA integrity. To date, few studies have investigated this phenomenon in horses, particularly in industry-relevant environments. Therefore, this study examined the relationship between ambient climatic conditions and fertility within a population of commercially fertile stallions. Post-coital semen samples (*n* = 804) were collected weekly from 46 Thoroughbred stallions during two successive breeding seasons (NSW, Australia; 22 weeks total). Semen samples were processed via single-layer colloidal centrifugation on-site to remove contaminants, seminal plasma and poor-quality sperm cells. The remaining sperm fraction was resuspended in Biggers, Whitten and Whittingham (BWW) medium for analyses, including sperm concentration, motility, and DNA damage. Fertility data (first cycle and per-cycle pregnancy rates) were collected from farms. Loggers were placed in the stables and paddocks of stallions to record ambient temperature and humidity, on 3-min cycles. Our results indicate that current management regimens, involving the stabling of stallions at night, with paddock access during the day, expose stallions to highest ambient climatic conditions. Polynomial distributed lag modelling identified 18 stallions whose fertility was adversely affected by heat stress, based on correlations between fertility and ambient climatic conditions (*p* ≤ 0.05). Of these 18 candidate stallions, six (13% of the overall population) exhibited repeated positive relationships between ambient climatic conditions and sperm DNA damage, and repeated negative relationships between fertility and sperm DNA damage (*r* ≥ 0.50; *p* ≤ 0.05). These results suggest that heat-induced subfertility was most likely mediated via oxidative damage to the sperm DNA, and that current management strategies should be assessed to prevent exposure of stallions to maximal climatic conditions.

**Supplementary Information:**

The online version contains supplementary material available at 10.1007/s00484-025-03123-y.

## Introduction

Systemic heat stress is detrimental to sperm production and male fertility, leading to decreased sperm motility (Argov-Argaman et al. 2013; Wach-Gygax et al. 2017); increased morphological abnormalities (Abdelhamid et al. 2019; Cruz Júnior et al. 2015; Rahman et al. 2011); and increased damage to DNA (Paul et al. 2009; Pérez-Crespo et al. 2008). Successful spermatogenesis typically requires temperatures 2 – 7 °C lower than the core body temperature to produce viable, intact spermatozoa (Neto et al. 2013; Waites 1991). Like most other mammals, stallions are equipped with several testicular thermoregulatory strategies, such as evaporative cooling (Waites 1970; Waites and Setchell 1964), and heat exchange (Cook et al. 1994; Dahl and Herrick 1959), that maintain testes approximately 3.5 °C below core body temperature. While these mechanisms function effectively during minor temperature fluctuations (30–32 °C; Neto et al. 2013), in regions such as the Hunter Valley, Australia―the second largest Thoroughbred breeding centre in the world―late spring, and early summer temperatures can exceed 40 °C, surpassing thermoregulatory capacities. This situation is compounded by the fact it aligns with the Thoroughbred breeding season, and that climate models forecast a temperature increase up to 2.3 °C within 50 years (NSW Office of Environment and Heritage 2014). Considering the magnitude of the Australian Thoroughbred breeding industry, this phenomenon must be thoroughly examined.

Several studies have also reported putative associations between stallion ejaculates collected during summer months and decreased sperm quality (Argov-Argaman et al. 2013; Wach-Gygax et al. 2017), increased lipid peroxidation, protein carbonylation, and DNA damage (Balić et al. 2012; Mathevon et al. 1998; Mislei et al. 2020; Wach-Gygax et al. 2017). Localised scrotal heating experiments in stallions—although not entirely reflective of whole-body heat insult—have indicated that testicular degeneration occurs in response to elevated temperatures, through decreased circulating testosterone concentrations, reduced sperm concentration, increased DNA damage, and a reduction in the percentage of morphologically normal, progressively motile spermatozoa (Blanchard et al. 1996; Love and Kenney 1999). Strikingly, sperm parameters do not return to normal levels until seven weeks post-heat insult (Blanchard et al. 1996).

In a murine model, ambient temperatures of 35 °C led to increased reactive oxygen species (ROS) generation, and a consequent rise in sperm oxidative DNA damage (Houston et al. 2018). While these damaged spermatozoa were fertile, sperm harbouring oxidatively damaged DNA can give rise to *de novo* mutations in the embryo, leading to embryo loss and developmental disease in the offspring (Casanovas et al. 2019; Ohno et al. 2014; Sakkas et al. 1998). Accordingly, the delivery of paternal heat-induced DNA damage to the oocyte may be a major driving factor of early embryo loss in horses (Allen 2001; Allen et al. 2007) and congenital abnormalities in foals (Crowe and Swerczek 1985; Galvin and Corley 2010; Giles et al. 1993). Whether the higher ambient temperatures experienced by stallions throughout the breeding season elicit oxidative sperm DNA damage or decreased fertility rates remains to be proven. As such, this study aims to examine the relationship among climatic conditions, DNA damage, and fertility within a population of commercially fertile breeding stallions.

## Materials and methods

### Experimental design, study population and management

This study investigated the relationship between ambient climatic conditions and fertility outcomes in a population of commercially fertile Thoroughbred stallions (*n* = 46), housed across four farms within a 200 km radius in the Hunter Valley region of Australia. During two consecutive breeding seasons, semen samples were collected once per week at each farm. Due to the prohibition of assisted reproductive technologies in Thoroughbred breeding, dismount semen samples were used, as they provide a representative sample of the entire ejaculate (Gibb et al. 2014) along with a matched breeding outcome. Semen samples were processed on-farm and assessed for DNA damage. Pregnancy scan results, pertaining to each breeding that each stallion performed, were provided by stud farms. From these data, relationships between ambient climatic conditions, stallion fertility, and sperm DNA damage were investigated.

Across all farms, stallions were managed under standard commercial breeding conditions, with access to pasture during the day (6 am – 4 pm) and stabled overnight. Stallions were typically bred twice daily, with a maximum of four breedings per day, as determined by individual farm managers. The selection of mares for breeding was made entirely by individual horse owners and farm managers. Mares were generally bred once per cycle; however, rebreeding within the same cycle occasionally occurred at the discretion of farm managers or veterinarians—most commonly when breeding success was uncertain, the mare had a history of subfertility, or ovulation had not yet occurred. Rebreeding was only performed when mares were confirmed to still be in oestrus. Of the samples collected in this study (*n* = 804), 8.5% were associated with re-breeding events, and were retained in the analysis to reflect common industry practices.

### Reagents

Research-grade chemical reagents were obtained from Sigma-Aldrich (Castle Hill, NSW, Australia) unless otherwise specified. A modified Biggers, Whitten and Whittingham medium (Biggers et al. 1971) was used to prepare and incubate spermatozoa for analyses, and contained 95 mM NaCl, 4.7 mM KCl, 1.7 mM CaCl_2_.2H_2_O, 1.2 mM KH_2_PO_4_, 1.2 mM MgSO_4_.7H_2_O, 25 mM NaHCO_3_, 5.6 mM D-glucose, 275 mM sodium pyruvate, 3.7 µL/mL 60% sodium lactate syrup, 50 U/mL penicillin, 50 mg/mL streptomycin, 20 mM HEPES (GE Healthcare, Parramatta, NSW, Australia) and 0.1% (w/v) polyvinyl alcohol, with an osmolarity of 310 mOsm/kg and a pH of approx. 7.2.

### Sample collection and preparation

Institutional and New South Wales State Government ethical approval was secured for the use of biological material and procedures in this study (approval number: A-2011-122). Post-breeding equine dismount semen samples (*n* = 804 samples) were collected from 46 stallions across four commercial Thoroughbred stud farms associated with the Hunter Valley Equine Research Centre, Australia. Samples were obtained over 11 consecutive weeks during each of two consecutive breeding seasons (commencing 11-September in both years; *n* = 486 Year 1, and *n* = 318 Year 2). Collection required residual semen to be gently milked from the urethra, directly into a sterile specimen container as the stallion dismounted the mare. Semen was immediately transferred into a prewarmed (37 °C) 15 or 50mL Falcon tube and diluted (2:1, extender: semen) with EquiPlus extender (Minitube Australia, Ballarat, VIC, Australia). Semen samples were processed via single-layer colloidal centrifugation on-site using EquiPure gradients (Tek-Event, Round Corner, NSW, Australia; 500 × g, 20 min) to isolate high-quality spermatozoa (Griffin et al. 2020). Sperm pellets were resuspended in BWW and maintained at room temperature (RT) for analyses.

### Motility analysis

Sperm motility was objectively determined on-farm using an iSperm device (version 4.5.2; Aidmics Biotechnology Co., LTD, Taiwan) according to manufacturer’s instructions. Each sample was assessed in triplicate, using the supplied base and cover chips. Total motility (%), progressive motility (%), average path velocity (VAP, µM/s), curvilinear velocity (VCL, µM/s), straight-line velocity (VSL, µM/s), straightness (STR), and linearity were assessed. Cells exhibiting VAP ≥ 20µM/s and VSL ≥ 3µM/s were considered motile (total motility). Cells exhibiting VAP ≥ 50µM/s and STR ≥ 75% were considered progressive.

### DNA damage assessment

#### Oxidative DNA damage by flow cytometry

Samples collected during the first breeding season were assessed using the OxyDNA assay kit (Calbiochem, CA, USA) for oxidative guanine adducts (8-hydroxy-2′-deoxyguanosine [8OHdG]), as described by De Iuliis et al. (2009) with minor modifications. Spermatozoa were pelleted by centrifugation (500 × g, 5 min) and incubated in PBS containing 0.05% Triton X-100 and 1.95 mM 1,4-Dithiothreitol (DTT) for 10 min (RT) to permeabilise cells and relax chromatin. Cells were centrifuged (500 × g, 3 min), and fixed by resuspending the pellet in 100 µL of 2% paraformaldehyde and incubated for 10 min (4 °C). Cells were then washed in PBS (2000 × g, 2 min), and stored in 0.1 M glycine in PBS at 4 °C. On the day of assessment, sperm pellets were resuspended in 100 µL of permeabilisation solution (3.4 mM sodium citrate, 0.1% Triton X-100 in 1× PBS) and incubated in the dark (4 °C) for 5 min. Cells were centrifuged (2000 × g, 3 min), and resuspended in a 1:50 dilution of charcoal-treated fluorescein isothiocyanate (FITC)-conjugate solution, using the provided wash solution (200 µL OxyDNA assay kit wash solution in 4800 µL Milli-Q water [Millipore, Australia]) and incubated for 1 h in the dark (37 °C). Cells were centrifuged (2000 × g, 2 min) and resuspended in 200 µL PBS and analysed using a FACSCanto II flow cytometer (Becton Dickinson, NJ, USA), with a 488-nm solid state and 633-nm HeNe laser, and classified according to their 8OHdG positivity (green fluorescence intensity). Emission measurements were made using 530/30-nm band pass (green/FITC) and 660/20-nm band pass (red/APC) filters. Forward scatter and side scatter dot plots were used to gate sperm cells and exclude contributions from contaminating cells and/or debris. All data were acquired and analysed using FACSDiva software v8.01 (Becton Dickinson, Franklin Lakes, NJ, USA) with a minimum of 10,000 events/sample.

#### Combined sperm chromatin dispersion (‘Halo’) and oxidative DNA damage assay (8OHdG)

As a complementary DNA integrity measure, samples collected during the second breeding season were assessed using a novel combination of the oxidative DNA damage (8OHdG) assay and the sperm chromatin dispersion (‘Halo’) assay, as described by Houston et al. (2019). Briefly, spermatozoa were snap-frozen in liquid nitrogen on-site, and stored at −80 °C until analysed. On the day of assessment, cells were thawed over ice, mixed with 1.0% low melting agarose (Bio-Strategy, Tingalpa QLD, Australia; 7:3, agarose: sperm). A 70 µL aliquot of the sperm-agarose solution was spread evenly across a prewarmed (35 °C) microscope slide, pre-coated with 0.65% agarose. Slides were sealed with a coverslip and incubated (4 °C, 10 min). Coverslips were removed and the slides were treated (RT) with HCl (0.08 N) for 7 min, followed by ‘Halo solution 1’ (0.4 M Tris, 1% SDS, 50 mM EDTA, 0.8 M DTT, pH 7.5) for 20 min, and ‘Halo solution 2’ (0.4 M Tris, 1% SDS, 2 M NaCl, pH 7.5) for 5 min to lyse the cells, and to relax and neutralise the DNA. Slides were then treated with Tris-boric acid-EDTA buffer (TBE; 0.1 M tris, 0.09 M boric acid, 0.002 M EDTA, pH 8.0) for 2 min (RT), and washed with increasing concentrations of ethanol (70, 90, and 100%) for 2 min each (RT). Slides were allowed to airdry for 15 min, before incubating overnight in a humid chamber (4 °C) with a DNA/RNA damage antibody (1:50; In Vitro Technologies, Noble Park North, VIC, Australia). Slides were washed (2 × 5 min, PBS), and incubated with Alexa Fluor488 secondary antibody (Thermo Fisher Scientific; 1:400 in PBS) in a humid chamber (90 min, RT). Slides were counterstained with the nuclear marker, 4′,6-diamindino-2-phenylindole (DAPI; 1:2000 in PBS) for 2 min (RT) and rinsed with PBS (×2) to remove unbound stain. Mowiol mounting medium was placed on the slides before sealing with a coverslip. Slides were imaged using an AXIO Imager.A1 fluorescence microscope (Carl Zeiss Micro Imaging GmbH, Jena, Thuringia, Germany). Imaging parameters were kept consistent between groups, fluorescence intensity was assessed against a secondary antibody-only control using ImageJ software (Schindelin et al. 2012). Thresholds were adjusted to measure the mean fluorescence intensity and total area of both the inner nucleoid and the ‘halo’.

### Fertility rates

Pregnancy was confirmed via transrectal ultrasonography, conducted by a reproductive veterinarian, 12 to 14 days after ovulation. Pregnancy scan results were provided by stud farms, at the end of each breeding season. From these data, daily and 7-day mean first cycle pregnancy (FCP) rates and per-cycle pregnancy (PCP) rates were calculated for each stallion. Mares that returned two or more successive negative pregnancy diagnoses, without any subsequent positive diagnosis, were characterised as ‘low fertility mares’, and were removed from the PCP rate calculations.

### Climatic data

USB Temperature and Humidity Data Loggers (Jaycar, Rydalmere, NSW, Australia) were installed in the stable barns in which stallions were residing, and in a paddock central to where stallions were grazing. Loggers were installed out of direct sunlight to prevent exposure to solar radiation and thereby false measurements. Stable loggers were installed on opposing walls of each stable, out of reach from stallions and not lying flat against surfaces. Loggers were calibrated using a mercury thermometer and programmed to record temperature (^○^C) and relative humidity (%) on a three-minute cycle. Data were downloaded using the provided software and transferred to Excel for analyses. Temperature humidity index (THI) was calculated using the following formula:

$$\%\mathrm{THI}\;=\;(0.8\mathrm T)\;+\;(\mathrm{RH}\;(\mathrm T\;-\;14.4))\;+\;46.4$$

*where T = ambient temperature in °C*,* RH = relative humidity expressed as a proportion.*

Temperature and THI hourly means were calculated (using data recorded between 1 and 59 min past the hour). These were used to determine the following variables: maximum temperature (Tmax); maximum THI (THImax); minimum temperature (Tmin); and minimum THI (THImin), either for the defined periods that stallions were in paddocks (6 am and 4 pm), or stabled (4 pm through to 6 am; Experiment 1), or for the entire day (12:00 am – 11:59 pm; Experiment 2). For the defined time period variables (Experiment 1), mean temperature (Tmean) and THI (THImean) were also calculated.

### Statistical analyses

#### Experiment 1: Comparison of paddock and stable climatic conditions

Comparisons of climatic variables recorded in stables and paddocks (Tmax; THImax; Tmean; THImean) were conducted for the defined periods that stallions were in paddocks (classified as ‘daytime’) and in stables (classified as ‘night-time’) across four farms. Pairwise comparisons were conducted using independent samples Student’s t-tests for each calendar day. Statistical analyses were performed using SPSS (version 27), with significance set at *p <* 0.05.

#### Experiment 2: Effect of ambient temperature and THI on fertility and DNA damage

Univariate binary logistic regression models were fitted against daily fertility rates; with the proportion of daily successful breedings (weighted for number of breedings each day) as the outcome variable, and a single explanatory variable chosen from the list below:

- Stable Tmax
- Stable THImax
- Paddock Tmax
- Paddock THImax
- Stable Tmin
- Stable THImin
- Paddock Tmin
- Paddock THImin

These regression models formed the foundation for investigating the associations between daily breeding success and past climatic conditions. We then fit 41 univariate models—each with a different time lag (0 – 40 days previous)—for each climatic variable, Briefly, in the 1^st^ model, a stallion’s daily fertility rate (outcome variable) was compared to the chosen climatic variable recorded on the same day; in the 2^nd^ model, the stallion’s daily fertility rate was compared to the climatic variable recorded one day prior. This continued up to the 41^st^ model (Table 1).

**Table 1:** Example models used to explore the associations between stallion fertility and past climatic conditions

Model	Example Climatic Variable	Recorded
1^st^	Maximum paddock temperature	Same day (day 0)
2^nd^	Maximum paddock temperature	1 day prior (day 1)
3^rd^	Maximum paddock temperature	2 days prior (day 2)
….		
41^st^	Maximum paddock temperature	40 days prior (day 40)

The 41 regression coefficients, and associated 95% confidence intervals, were saved, and used to graph breeding success (on the scale of the linear predictor) against the regression coefficient at each time lag. Graphs were examined to identify the significant regression coefficients that suggested a possible relationship between breeding success on the current day with the ambient climatic variable at that time lag. Analyses were separately undertaken for daily PCP rate and FCP rate recorded by each stallion. Model fitting was carried out using R (Version 4.0.2., R Core Team 2016) via the generalised linear model function.

## Results

### Experiment 1: Comparison of paddock and stable climatic conditions

Across all four farms, stallions typically had access to paddocks between 6 am and 4 pm (classified as ‘daytime’) and were stabled from 4 pm through to 6 am (classified as ‘night-time’). Following the cessation of each breeding season, temperature and humidity data recorded on each farm were collated, and the temperature humidity index (THI; a combined measurement of temperature and humidity, and indicator of heat load risk) was calculated.

**Fig. 1:**
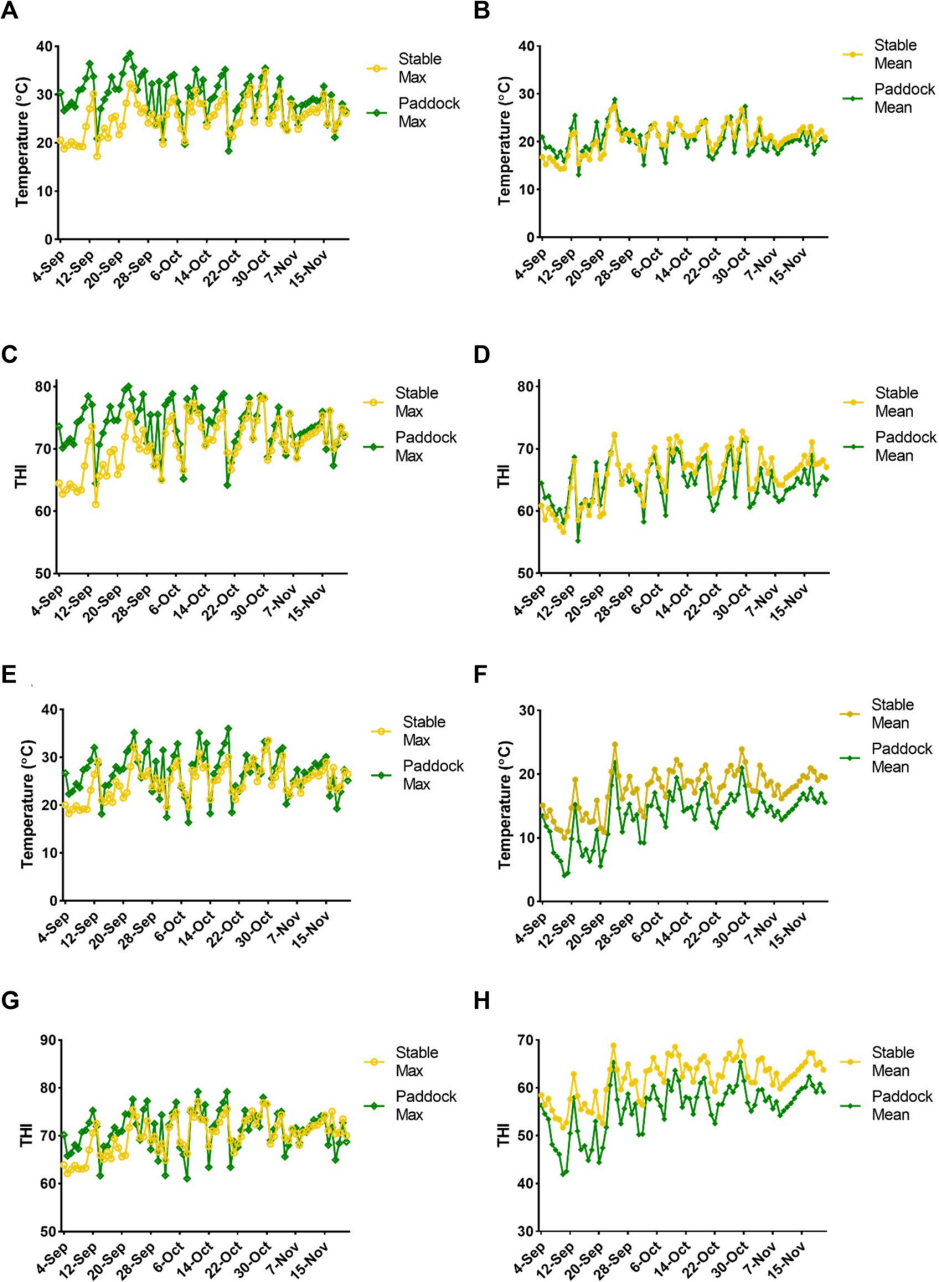
Comparison of daytime (**A **– **D**) and night-time (**E **– **H**) climatic conditions between stables and paddocks, recorded throughout the first breeding season. Daytime maximum temperature (**A**), mean temperature (**B**), maximum temperature humidity index (THI; **C**) and mean THI (**D**) of stables and paddocks. Night-time maximum temperature (**E**), mean temperature (**F**), maximum THI (**G**) and mean THI (**H**) of stables and paddocks

Paddock Tmax and THImax recorded during ‘daytime’ hours (when stallions are grazing), were typically higher than stable Tmax and THImax, for most of the recorded breeding season (94% and 81% of the season for paddock Tmax and THImax, respectively; Fig. 1A, C)—exposing stallions to maximal temperature and THI. In contrast, differences between ‘night-time’ Tmax and THImax of stables and paddocks were relatively similar throughout the breeding season, only reached significance on seven (9.0%) and five (6.0%) days, respectively (Fig. 1E, G; Supplementary Tables 3, 4, 5; *p* ≤ 0.05). However, ‘night-time’ stable Tmean and THImean were consistently higher than those of paddock measurements, reaching statistical significance on 29 (36.7%) and 43 (54.4%) days, respectively (Fig. 1F, H, Supplementary Table 3; *p* ≤ 0.05)—exposing stallions to higher mean temperature and THI during the night.

### Experiment 2: Effect of ambient temperature and THI on fertility and DNA damage

**Fig. 2:**
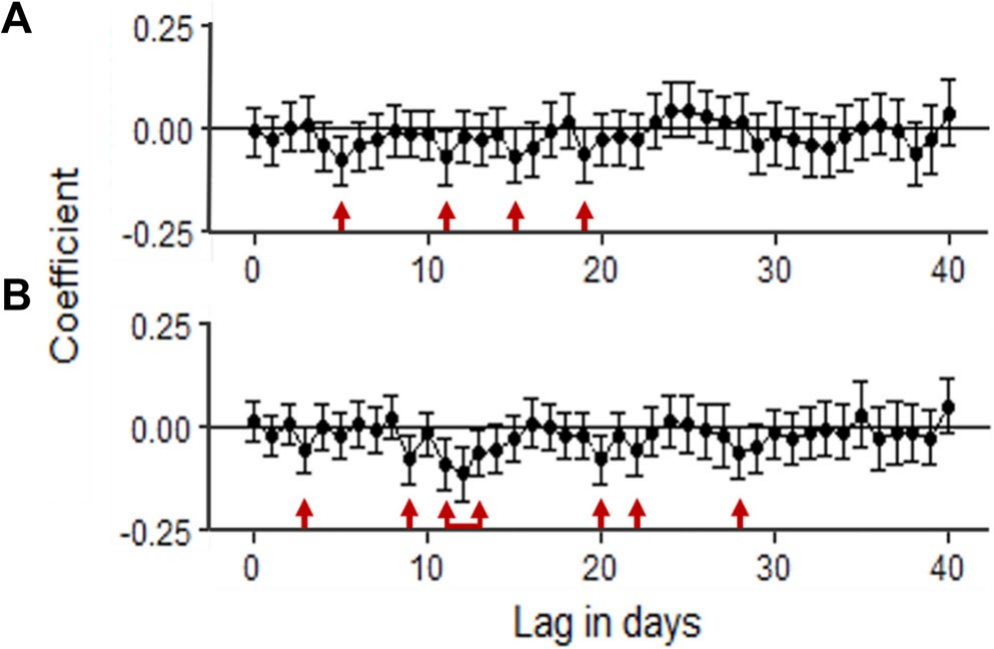
Identifying times at which stallion fertility is negatively affected following increased ambient conditions. In this example, plotting the correlations revealed that the fertility (PCP rate) of Stallion 46 was significantly negatively impacted by maximum paddock temperature at days 5, 11, 15 and 19 post-heat event (**A**), whereas the fertility (PCP rate) of Stallion 24 was significantly negatively impacted by maximum stable temperature at days 3, 9, 11–13, 20, 22 and 28 post-heat event (**B**); Cl = 95%, *p* ≤ 0.05

Herein, we aimed to assess whether the fertility fluctuations of individual stallions occurred in response to climatic conditions. Using polynomial lag modelling, Pearson correlations were carried out in a sequential manner, comparing daily fertility rates with climatic measures (Table 1) recorded on that day, and consecutively up to 40 days prior. For these experiments, Tmax and THImax refers to the highest values recorded in a 24-hour period (12:00 am – 11:59 pm). A total of 16 univariate logistic regression models (eight climatic variables × two outcome variables: PCP and FCP) were fitted for each horse. We then plotted these correlations to identify at what points fertility was negatively affected following a heat event. For example, Fig. 2 illustrates the relationship between fertility and paddock Tmax (Fig. 2A) and stable Tmax (Fig. 2B) for two stallions. The fertility of Stallion 24 is significantly negatively affected by paddock Tmax at 5, 11, 15 and 19 days following a ‘heat event’ (*p* ≤ 0.05; Fig. 2A). A heat event is defined as the initial change in climatic conditions (paddock Tmax and/or THImax) that is associated with a significant negative correlation with stallion fertility. In contrast, the fertility of Stallion 46 is significantly negatively affected by stable Tmax on days 3, 9, 11–13, 22 and 28 post-heat event (*p* ≤ 0.05; Fig. 2B).

**Fig. 3:**
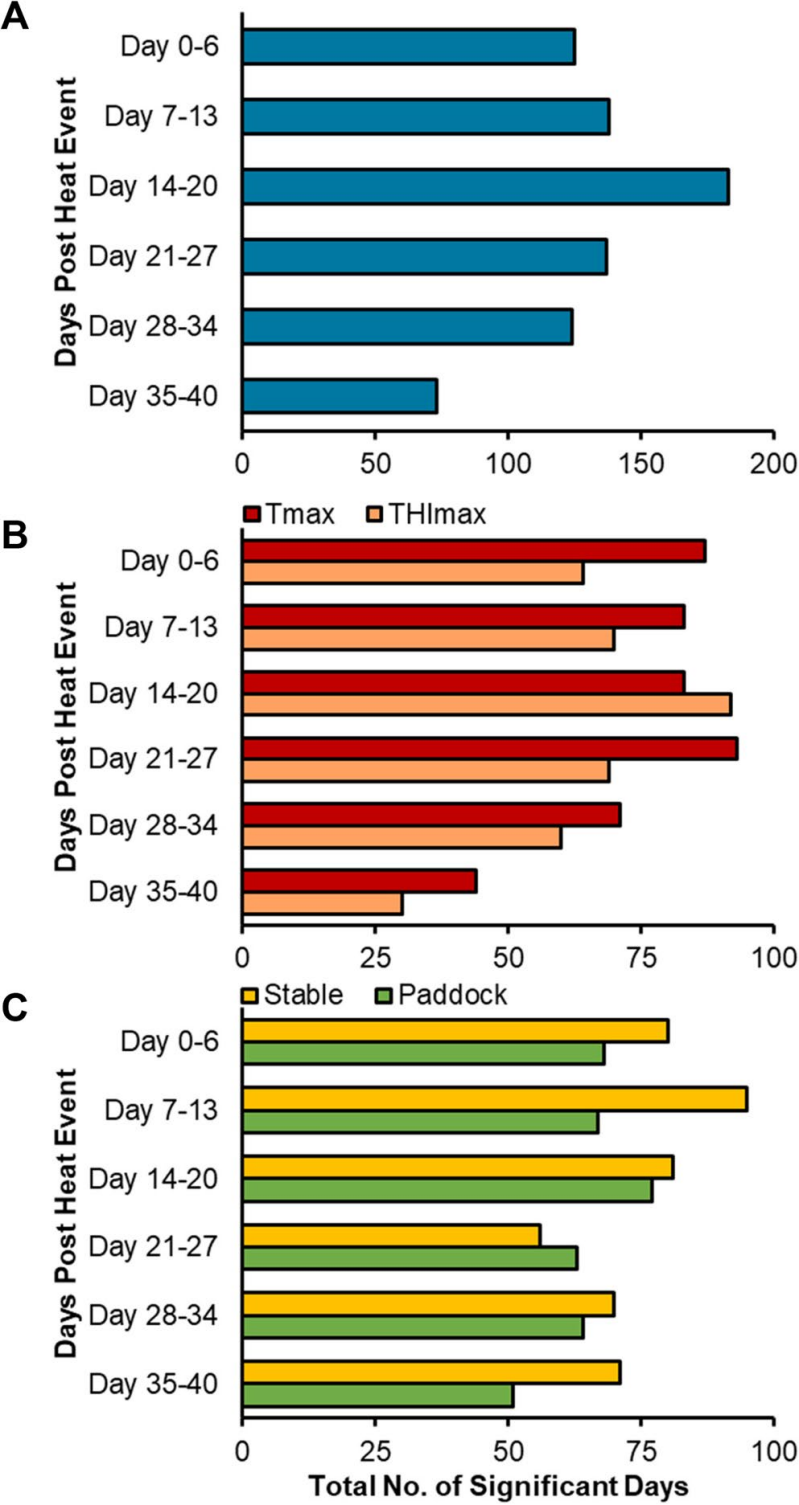
Identifying the most frequent periods where stallion fertility is negatively affected by increased ambient conditions. Pearson correlations were carried out comparing stallion fertility with climatic parameters (maximum paddock and stable, temperature [Tmax] and THI [THImax]) recorded on the corresponding day, and subsequently up to 40 days prior. This analysis was carried out on all stallions, and days where fertility was negatively influenced by climatic conditions (*p* ≤ 0.05) were logged **(A)**. Data were then grouped by **B**) environmental measure (Tmax vs. THImax) and **C**) location to identify when the greatest effects on stallion fertility were occurring

Using these data, we then tallied the number of times fertility was significantly negatively affected by climatic conditions on a specific day (day 0 – day 40) across all stallions, and grouped these data in 7-day intervals. We identified that Tmax and THImax exhibited greatest effects on fertility 14 – 20 days following a heat event, however, most other date categories were well represented (Fig. 3A). Separating and comparing these data by climatic conditions (Fig. 3B) and location (Fig. 3C) provides an insight into how these factors influence fertility.

By totalling the number of occasions the fertility of each stallion was significant negatively affected by Tmax or THImax, we identified a sub-population of 18 candidate heat-susceptible stallions (Table 2); accounting for 40% of the studied population. These stallions experienced a minimum of 20 days where fertility (both PCP and FCP rate) was significantly negatively affected by ambient climatic conditions. To confirm our methodology, we repeated these analyses, this time using the minimum stable and paddock temperature (Tmin) and THI (THImin) recorded throughout the entire day (12:00 am – 11:59 pm). We hypothesised that a similar cohort of stallions would demonstrate repeated heat-sensitivity, due to temperature and THI failing to drop significantly, and as such, failing to provide animals with a recovery period. Studies in cattle have shown that a reduction in overnight heat load is essential in enabling these animals to withstand elevated daytime climatic conditions (Gaughan et al. 1999, 2023; Hahn 1999; Mader 2003). In total, six stallions experienced a minimum of 12 days where fertility was significantly negatively affected by minimum ambient climatic conditions (Tmin and THImin). All bar one of these stallions appeared in our initial cohort of 18 candidate heat-susceptible stallions, thereby confirming our hypothesis and methodology.

**Table 2:** Findings of the correlation analyses conducted on the 18 candidate heat-affected stallions

Stallion	No. of Days that Fertility was Negatively Affected		No. of Monitored Breeding Seasons	Farm	Age During Year 1 (years)	Fertility and DNA Damage Significant Correlations (*p ≤* 0.05)				Environmental Conditions and DNA Damage Significant Correlations (*p* ≤ 0.05)							
	Tmax and THImax	Tmin and THImin				8OHdG^1^ × PCP Rate (Year 1)	8OHdG^1^ × FCP Rate (Year 1)	DNA Damage^2^ × PCP Rate (Year 2)	DNA Damage^2^ × FCP Rate (Year 2)	8OHdG^1^ × Pdk Max (Year 1)	8OHdG^1^ × Stb Max (Year 1)	8OHdG^1^ × Pdk Mean (Year 1)	8OHdG^1^ × Stb Mean (Year 1)	DNA Damage^2^ × Pdk Max (Year 2)	DNA Damage^2^ × Stb Max (Year 2)	DNA Damage^2^ × Pdk Mean (Year 2)	DNA Damage^2^ × Stb Mean (Year 2)
46	80	28	1	1	≤ 8	―	―	Yes (Halo only)	Yes (Halo only)	―	―	―	―	Yes	Yes	Yes	No
25	45	0	1	2	9–15	Yes (Low)	Yes	―	―	Yes (Low)	No	No	Yes (Low)	―	―	―	―
10	37	0	2	3	9–15	Yes (Low)	Yes (Low)	―	―	Yes (Low)	No	Yes (Low)	No	―	―	―	―
39	36	0	1	1	9–15	Yes (Low)	No	―	―	Yes (Low)	No	No	No	―	―	―	―
9	38	58	2	3	≤ 8	Yes	Yes (Low)	Yes (Halo only)	Yes (Halo only)	Yes	No	Yes	No	Yes	Yes	Yes	Yes
42	32	12	2	1	≤ 8	No	No	No	No	No	No	No	No	No	No	No	No
41	28	4	2	1	9–15	No	No	No	No	No	Yes	No	Yes	Yes (Low)	Yes	Yes (Low)	Yes
30	27	5	1	2	≤ 8	―	―	Yes (Low)	No	―	―	―	―	Yes (Low)	No	No	No
6	21	0	2	3	9–15	No	No	No	No	No	Yes (Low)	No	Yes (Low)	Yes (Low)	Yes (Low)	Yes (Low)	Yes
3	40	24	2	3	≤ 8	No	No	Yes (Low)	Yes (Low)	Yes	No	Yes	No	Yes	Yes	Yes	Yes
13	31	6	1	3	≤ 8	―	―	Yes (Halo only)	Yes (Halo only)	―	―	―	―	Yes	Yes	Yes	Yes
27	31	0	1	2	≤ 8	Yes	Yes (Low)	―	―	Yes	No	No	No	―	―	―	―
4	25	5	2	3	≤ 8	No	No	No	No	No	No	Yes	No	Yes	Yes	Yes	Yes
36	24	18	2	4	≤ 8	Yes	Yes	Yes (8OHdG only)	Yes (8OHdG only)	Yes	Yes	Yes	No	Yes (8OHdG only)	Yes (8OHdG only)	Yes (8OHdG only)	No
34	22	1	2	4	≥ 16	Yes	Yes	Yes (8OHdG only)	Yes (8OHdG only)	No	No	No	No	Yes (8OHdG only)	Yes (8OHdG only)	Yes (8OHdG only)	Yes (8OHdG only)
7	21	0	2	3	≥ 16	No	No	―	―	Yes	Yes (Low)	Yes	No	―	―	―	―
18	20	1	2	2	≤ 8	Yes	Yes	No	No	Yes	No	Yes	No	Yes	Yes	Yes	Yes
37	23	2	2	4	≤ 8	Yes (Low)	Yes (Low)	No	No	No	No	No	No	Yes	Yes	Yes	Yes

where PCP rate: per-cycle pregnancy rate; FCP rate: first cycle pregnancy rate; T: temperature; THI: Temperature Humidity Index; Max: maximum temperature and THI; Min: minimum temperature and THI; Mean: mean temperature and THI; Stb: stable; Pdk: paddock; 8OHdG: 8-hydroxy-2′-deoxyguanosine; Halo only: correlating with combined sperm chromatin dispersion and 8OHdG assay only; 8OHdG only: correlating with 8OHdG fluorescence only; Low: *r* < 0.5 and/or not consistent across all variables assessed

^1^ Assessments carried out using the OxyDNA assay kit

^2^ Assessments carried out using the combined sperm chromatin dispersion (‘Halo’) and 8OHdG assay

**Fig. 4:**
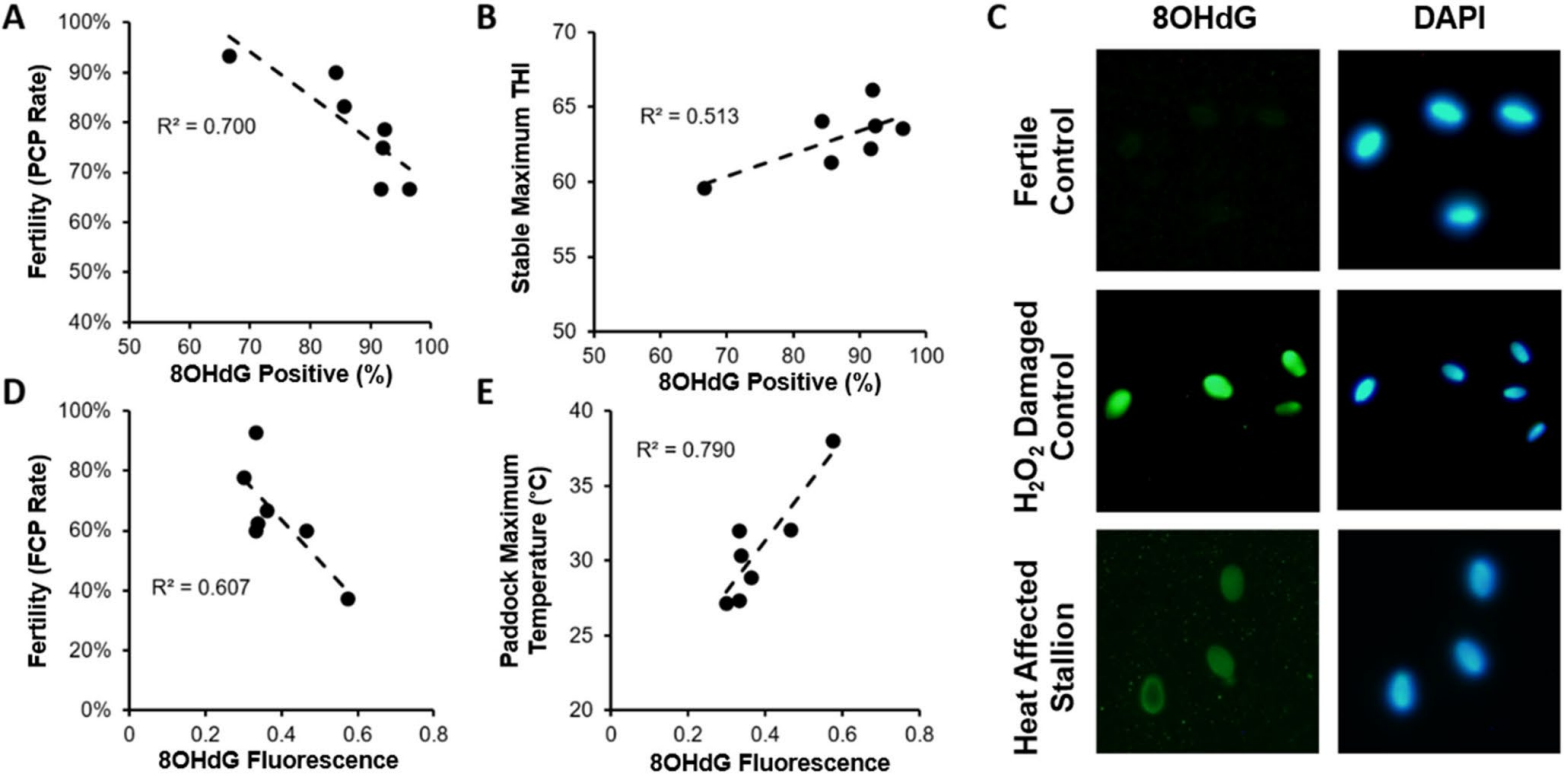
The relationship between climatic conditions, sperm DNA damage, and fertility of Stallion 36. Oxidative DNA damage of samples collected during the first breeding season was assessed using an OxyDNA assay kit. Pearson correlations demonstrate relationships between the percentage of 8OHdG positive cells and per-cycle pregnancy (PCP) rate (**A**), and maximum stable temperature humidity index (THI; **B**). The DNA fragmentation and oxidative DNA damage of spermatozoa collected during the second breeding season were assessed using a combined sperm chromatin dispersion (‘Halo’) and oxidative DNA damage assay (8OHdG); *p* ≤ 0.05. Representative images of 8OHdG fluorescence and halos (DAPI) of spermatozoa collected from Stallion 36, a fertile control stallion and a hydrogen peroxide treated control (damaged control; **C**). Pearson correlations demonstrate relationships between 8OHdG fluorescence and first cycle pregnancy (FCP) rate (**D**), and maximum paddock temperature (**E)**

Followup DNA damage assessments of sperm samples collected from this sub-population were carried out (Table 2; Fig. 4). These investigations revealed that, of the 18 stallions identified, six stallions demonstrated strong, negative relationships between both DNA damage and fertility, and DNA damage and climatic conditions (Table 2)—accounting for 13% of the entire studied population. To explore this effect, and to demonstrate how investigations into each stallion were carried out, we present the findings of Stallion 36 as a case study.

### Case study: Stallion 36

Stallion 36 presented repeated strong statistically significant relationships between fertility, ambient climatic conditions, and DNA damage assessments across both breeding seasons. This Australian-born stallion was five years old at the commencement of this study. Table 3 details the weekly fertility (PCP and FCP) rate of Stallion 36 over the first and second breeding seasons. This stallion’s fertility was significantly negatively influenced by maximum climatic conditions (Tmax and THImax) on 24 occasions, and by minimum climatic conditions (Tmin and THImin) on 18 occasions. Consistent with our findings presented in Fig. 3, the fertility of Stallion 36 was most frequently negatively impacted 11 – 19 days following an initial heat insult. Intriguingly, Stallion 36’s fertility was also negatively affected immediately post-heat insult (days 0 – 1); 4 – 6 days post; ~five weeks (day 29) post; and six weeks (days 38 – 39) post-heat insult. Detailed assessment of this stallion’s sperm DNA integrity using the ‘Halo’ assay (assessing level of DNA fragmentation), alongside the oxidative DNA damage assays (8OHdG; Fig. 4) indicated that Stallion 36 exhibited strong, negative relationships between oxidative DNA damage and fertility (PCP rate and FCP rate; Fig. 4A, D; Table 4). In addition, this stallion exhibited strong, positive relationships between oxidative DNA damage and climatic conditions (Tmax and THImax; Fig. 4B, E; Table 4).

**Table 3:** Weekly per-cycle pregnancy (PCP) rate and first cycle pregnancy (FCP) rate recorded by Stallion 36 throughout the studied breeding seasons

Year	Week Starting	PCP Rate	No. Bred	FCP Rate	No. Bred
1	2-Sept	70.0%	10	63.6%	11
	9-Sept	83.3%	6	83.3%	6
	16-Sept	83.3%	6	83.3%	6
	23-Sept*	86.7%	15	73.3%	15
	30-Sept*	66.7%	6	80.0%	5
	7-Oct*	66.7%	12	50.0%	14
	14-Oct*	78.6%	14	75.0%	12
	21-Oct*	54.5%	11	50.0%	6
	28-Oct	90.0%	10	88.9%	9
	4-Nov*	77.8%	9	85.7%	7
	11-Nov	66.7%	6	50.0%	4
	18-Nov*	63.6%	11	50.0%	8
	25-Nov	100.0%	3	N/A	0
2	1-Sept	72.7%	11	64.3%	14
	8-Sept	60.0%	10	63.6%	11
	15-Sept*	60.0%	10	46.2%	13
	22-Sept	60.0%	10	62.5%	8
	29-Sept	66.7%	15	58.3%	12
	6-Oct*	91.7%	12	88.9%	9
	13-Oct*	83.3%	12	55.6%	9
	20-Oct	75.0%	12	88.9%	9
	27-Oct*	63.6%	11	75.0%	12
	3-Nov*	20.0%	5	33.3%	6
	10-Nov	81.8%	11	77.8%	9
	17-Nov*	60.0%	5	60.0%	5
	24-Nov	80.0%	10	66.7%	6

where PCP: Per-cycle pregnancy rate (low-fertility mares removed); FCP: first cycle pregnancy rate; No. Bred: number of individual breedings

*Samples assessed for oxidative DNA damage

**Table 4:** Relationships presented by Stallion 36 between per-cycle pregnancy (PCP) rate and first cycle pregnancy (FCP) rate, climatic conditions, and DNA damage assessments

DNA Damage Results	Fertility		Climatic Conditions	
	PCP Rate	FCP Rate	Tmax	THImax
Year 1 Samples	−0.548	−0.837	0.919	0.778
Year 2 Samples	−0.779	−0.747	0.889	0.837

where Tmax: Maximum temperature (°C); THImax: Maximum Temperature Humidity Index. Pearson correlation, *p* ≤ 0.05

## Discussion

This is the first study to investigate the effects of ambient climatic conditions on stallion fertility in a commercial setting. We have shown that the standard management regimens (of stabling stallions at night with access to paddocks during the day) are exposing stallions to unfavourable climatic conditions, which may adversely affect their fertility. We also identified a sub-population of six stallions (13% of the studied population) who appear to be particularly susceptible to heat stress, displaying periods of reduced fertility alongside increased sperm oxidative DNA damage.

Most commercial stud farms stable stallions at night and allow paddock access during the day. Herein, we have demonstrated that these practices expose stallions to unfavourable climatic conditions, as paddock daytime measurements consistently peak above those of stables (Fig. 1A, C). At night, even though peak measurements are comparable (Tmax, THImax; Fig. 1E, G), mean nightly conditions are consistently higher in stables, indicating that stables retain more heat, subjecting stallions to warmer conditions than would be experienced outdoors. We hypothesise that a change in management regimens—allowing stallions access to pasture overnight when ambient temperature and THI has dropped (with stabling during the day)—would dramatically reduce exposure to maximal climatic conditions. Little research has been conducted into effective cooling strategies (e.g., misting systems, air conditioning) for equine housing. We observed that the greatest differences between daytime stable and paddock conditions (Tmax and THImax; Supplementary Tables 1, 2) occur in the first third of the breeding season; thereafter, significance is largely lost. This suggests that as heat rises over the season, stables become less effective at providing effective cooling. In the face of rising global temperatures, this is an area that requires immediate attention.

THI was developed as an indicator of heat load risk, accounting for the combined effects of temperature and relative humidity on livestock health (Brouk et al. 2003; Thom 1959), and has been widely used to assess transportation and housing conditions. (Atkins et al. 2018; Brouk et al. 2003; Gaughan et al. 1999; Osei-Amponsah et al. 2020). The index comprises five categories; cattle residing in conditions where THI ≥ 80 experience ‘moderate to severe heat stress’, as respiratory rates exceed 85 BPM and body temperatures exceed 40 °C (Atkins et al. 2018; Brouk et al. 2003; Osei-Amponsah et al. 2020). In the present study, of the 79 days monitored, daytime paddock THImax was ≥ 70 on 68 days (84.8%), and ≥ 75 on 32 days (40.5%; Fig. 1C), with one farm recording a paddock THImax ≥ 80 on 35 days (44.3%). An equine-specific THI scale has not yet been developed; however, it is conceivable that conditions inducing adverse physiological states in cattle will also deleteriously affect stallions. Further research is required to determine the THI levels causing discomfort and physiological stress in horses.

Spermatogenesis in the stallion lasts approximately 57 days, followed by a further seven days of epididymal storage (Johnson et al. 1997). Heat related insults, whether via systemic stress or direct, affect germ cells differently depending on their developmental stage at the time of insult (Houston et al. 2018; Netherton et al. 2022). As such, the timing of heat-induced subfertility can inform on the stages of germ cell development most vulnerable to damage (Love and Kenney 1999). While the entire spermatogenic and epididymal cycle spans 64 days, we limited our analysis window to the 40 days prior to semen collection for two main reasons. Firstly, previous equine studies demonstrating that heat stress can impair sperm parameters within two weeks and that these effects persist for several weeks—well within the 64-day cycle (Blanchard et al. 1996; Love and Kenney 1999). Secondly, due to the relatively short Thoroughbred breeding season (September to December), this window also allowed us to avoid bias introduced by analysing only late-season fertility data, when mare quality typically declines.

Herein, we demonstrated that the greatest effects on fertility are evident 14 – 20 days post-heat event (Fig. 3)—corresponding to spermatozoa in elongated and round spermatid stages of development. This is in line with published literature suggesting round spermatids and pachytene spermatocytes are particularly vulnerable to damage from stressors (Houston et al. 2018; Netherton et al. 2022; Pérez-Crespo et al. 2008), and with Love and Kenney (1999) who reported an initial rise in stallion sperm DNA damage at 10 and 24 days post-heat treatment. However, periods before and after this timeframe were also highly represented (Fig. 3) and so are equally important when considering heat-induced fertility losses. In equines, the earliest stage at which pregnancy can be detected is 12 – 14 days post-ovulation via ultrasonography. This pregnancy result also forms the basis from which stallion fertility is quantified; however, it does not account for conceptions lost prior to detection, nor for later-stage pregnancy losses. In these cases, the underlying cause—be it male or female—cannot be determined. As a result, this method may underestimate the true extent of subfertility or embryonic compromise, particularly in the context of heat-induced DNA damage.

Spermatozoa have an abundance of substrates such as DNA, proteins, and unsaturated fatty acids, rendering them particularly susceptible to oxidative damage (Aitken et al. 2010; Jones and Stewart 1979; Shiva et al. 2011). Therefore, it is not surprising that stallion fertility may be adversely affected at any point following heat insult. Herein, we demonstrated that ambient temperatures can induce significant DNA damage (assessed via ‘Halo’ and 8OHdG) in our stallion cohort, as six of the 18 candidatate heat-affected stallions demonstrated strong, positive relationships between DNA damage and climatic conditions, and strong, negative relationships between DNA damage and fertility (Table 1). Such findings suggest that, in these stallions, heat-induced subfertility is most likely mediated via damage to sperm DNA. Regarding the remaining 12 stallions, it is unclear what mechanism is responsible for the reduction in fertility post-heat events. Previous studies have reported disruptions to mitochondrial membrane potential, and protein expression and function, associated with heat stress (Gong et al. 2017; Hamilton et al. 2016; Rao et al. 2016). Indeed, cytotoxic aldehydes such as 4-hydroxynonenal (4HNE) are produced in response to lipid peroxidation and inflict substantial damage to spermatozoa via their adduction, and subsequent degradation of vital sperm proteins (Bromfield et al. 2015; Griffin et al. 2020). Conceivably, one limitation of this study was the inability to conduct live cell assays on-site to assess the extent of reactive oxygen species (ROS) generation, lipid membrane status, and aldehyde generation throughout the breeding season. Similar future investigations are required to identify if such fertility losses in breeding stallions are mediated via lipid membrane or proteolytic damage.

In this study, dismount semen samples were processed using density gradient centrifugation to isolate the high-quality sperm fraction prior to analysis. This method selectively enriches for spermatozoa with greater motility, viability, and normal morphology—characteristics associated with a higher likelihood of fertilisation (Griffin et al. 2020; Guérin et al. 1989; Karamahmutoglu et al. 2014; Netherton et al. 2018)—thereby enabling assessment of DNA damage in the functionally relevant subset of sperm, rather than across the entire ejaculate. Of the six heat-affected stallions identified, we presented the case of Stallion 36 who displayed strong relationships between oxidative DNA damage, fertility, and ambient climatic conditions (Fig. 4; Table 4)—noting that these results are specific to the fertilisation-competent sperm fraction, and may not represent the full extent of damage across the entire ejaculate. Houston et al. (2018) eloquently demonstrated the cascade initiated by prolonged, high ambient temperature exposure (35 °C) on the increased production of ROS, and subsequent increase in oxidative DNA damage. Considering stallion spermatozoa rely heavily on mitochondrial OXPHOS for energy production, and consequently experience large basal amounts of ROS (Gibb et al. 2014), it is certainly conceivable that additional unchecked production of ROS could lead to the oxidised base adducts (8-oxo-7,8-dihydro2′-deoxyguanosine; 8OHdG) and the DNA fragmentation observed in this stallion (De Iuliis et al. 2009; de Souza-Pinto et al. 2001; Kasai 1997).

Despite the strong statistical relationships between heat events and fertility fluctuations across the season, Stallion 36’s weekly fertility rate was higher than industry means of around 60 – 65%, for most of the season (average PCP and FCP rate of 70% across year 1 and year 2; Table 3). Elevated levels of DNA damage do not necessarily translate to reduced fertility, but should these cells fertilise the oocyte, they may culminate in a compromised genome in the resulting embryo, potentially negatively impacting embryo development, or future offspring health (Aitken and Bakos 2021). Oxidised base residues can cause transversion mutations (G-C to T-A) in spermatozoa, which may alter gene expression and trigger *de novo* mutations if not repaired by the oocyte (Aitken 2017; Feng et al. 2003; Martin et al. 2019; Ohno et al. 2014). The persistence of DNA lesions and mutagenic bases could then increase the risk of embryo genetic and epigenetic abnormalities (Aitken 2017; Champroux et al. 2016; Dada et al. 2012), and embryo loss (Casanovas et al. 2019; Sakkas et al. 1998). As the DNA analyses conducted herein were performed on the fertilisation-competent sperm population, such genomic lesions may carry a greater likelihood of transmission to the embryo. Recent research in men has identified that specific regions outside of the histone- and protamine-packaged domains are especially vulnerable to oxidative attack (Xavier et al. 2019). This study revealed that oxidative damage occurs uniformly across the genome, with the exception of the sex chromosomes and chromosome 15; the latter being particularly vulnerable to damage (Xavier et al. 2019). Intriguingly, genes on human chromosome 15 are associated with a range of neuropsychiatric disorders (Janecka et al. 2019; Stefansson et al. 2008; Vazza et al. 2007); developmental disorders (Christian et al. 1999; Mignon-Ravix et al. 2007); and autoimmune diseases (Aitken et al. 2020; Dierlamm et al. 2003; Heerema et al. 2002). It would be of considerable interest to determine if specific regions of the stallion genome are particularly susceptible to heat-induced oxidative damage and if these regions might explain the propensity for early embryo loss readily documented in mares (Allen 2001; Allen et al. 2007). In addition, these data could determine if vulnerable chromosomes are known to house risk loci for certain conditions with a genetic risk component for the resultant foals.

Our investigations revealed that not all stallions are equally affected by heat stress. Some stallions did not present biologically meaningful relationships between climatic conditions, fertility, or DNA damage, whereas others presented consistent, strong relationships. This is in keeping with studies in cattle that have also reported differences in the heat tolerance of individual bulls (Netherton et al. 2022; Vogler et al. 1991, 1993; Walters et al. 2005). Netherton et al. (2022) reported that ambient temperatures greater than 34 °C resulted in poor sperm production in 63% of bulls assessed, with the spermatozoa of the remaining 37% unaffected. In recent years, greater focus has been placed on the inter-individual genetic variation that renders certain animals less heat tolerant than others (Carabaño et al. 2019; Gourdine et al. 2017; Kim et al. 2018; Liu et al. 2019). For instance, a novel single nucleotide polymorphism of the *ATP1A1 *gene is associated with heat tolerance traits in dairy cows (Liu et al. 2011). Similarly, stress tolerant rats display a significant upregulation of heat shock protein 70, and increased expression of transcription factor heat shock factor-1 (Jain et al. 2014), while in Large White sows, heritable thermoregulation traits are responsible for higher heat tolerance (Gourdine et al. 2017). It is therefore conceivable that there is a genetic component predisposing certain stallions to reduced heat tolerance, and so future heat investigations should be approached in an entirely individualised manner.

In conclusion, this is the first study to investigate the effects of ambient climatic conditions on stallion fertility in an industry relevant setting. We revealed that current management regimens are sub-optimal, exposing stallions to maximal temperature and THI. We identified that the fertility of 40% of the total studied population is likely adversely affected by ambient climatic conditions, and in one third of this subpopulation, fertility losses were most likely mediated through oxidatively damaged DNA. Future research must focus on elucidating the regions of the genome that are particularly vulnerable to heat-induced oxidative damage—with the aim of determining the heritable genetic risk to offspring—and determining the underlying mechanisms governing fertility losses in the remaining population. Amid rising global temperatures, management practices (including paddock access and stabling schedules) and the use of climate control measures in barns, should be thoroughly assessed to ensure optimal breeding efficiency and horse welfare.

## Data availability

The datasets generated during and analysed within the current study are available from the corresponding author on reasonable request.

## Supplementary information

Below is the link to the electronic supplementary material.

Supplementary Material 1

Supplementary Material 2
