# Supplementary material for: The fertility of a sub-population of stallions is negatively affected by ambient climatic conditions, mediated through DNA damage
Source: Int J Biometeorol. 2026 Mar 9;70(3):89. doi: 10.1007/s00484-025-03123-y (PMC12971856; doi:10.1007/s00484-025-03123-y)
Supplement: Supplementary file 1 — Supplementary Material 1 [file 484_2025_3123_MOESM1_ESM.docx]

**The fertility of a sub-population of stallions is negatively affected by ambient climatic conditions, mediated through DNA damage.**

International Journal of Biometeorology

Griffin, R. A.*, Miller, K., Colyvas, K., Sheridan, A., De Iuliis, G., Aitken, R.J., Baker, M., Gibb, Z. & Swegen, A.

*HMRI Infertility and Reproduction Research Program, School of Environmental and Life Sciences, College of Engineering, Science and Environment, University of Newcastle, Australia; [roisin.griffin@newcastle.edu.au](mailto:roisin.griffin@newcastle.edu.au)

**Supplementary material:**

***Supplementary Table 1*** *Daytime maximum and mean temperature recorded in paddocks and stables during the 2017 breeding seasons (p ≤ 0.05, mean values ± S.E.M.)*

| Daytime Maximum Temperature (⁰C) | | | |
| --- | --- | --- | --- |
| Date | ***All farms*** | | ***p*-Value** |
|  | **Stable** | **Paddock** |  |
| 4-Sep | 20.5 ±0.50 | 30.4 ±0.98 | ***0.000*** |
| 5-Sep | 18.8 ±0.43 | 26.7 ±0.64 | ***0.000*** |
| 6-Sep | 19.5 ±0.44 | 27.4 ±0.82 | ***0.000*** |
| 7-Sep | 20.2 ±0.52 | 28.4 ±1.70 | ***0.007*** |
| 8-Sep | 19.6 ±0.64 | 27.5 ±1.66 | ***0.009*** |
| 9-Sep | 19.3 ±0.87 | 30.9 ±2.29 | ***0.006*** |
| 10-Sep | 19.2 ±0.81 | 31.2 ±2.01 | ***0.003*** |
| 11-Sep | 23.4 ±0.66 | 33.4 ±1.54 | ***0.002*** |
| 12-Sep | 27.1 ±0.77 | 36.4 ±1.74 | ***0.005*** |
| 13-Sep | 30.1 ±0.65 | 33.8 ±1.26 | *0.068* |
| 14-Sep | 17.2 ±0.34 | 20.9 ±2.05 | *0.179* |
| 15-Sep | 21.2 ±0.40 | 27.1 ±1.65 | ***0.024*** |
| 16-Sep | 23.0 ±0.40 | 29.0 ±1.80 | ***0.031*** |
| 17-Sep | 21.1 ±0.53 | 30.5 ±1.93 | ***0.007*** |
| 18-Sep | 25.1 ±0.78 | 33.7 ±1.64 | ***0.006*** |
| 19-Sep | 25.5 ±0.22 | 31.2 ±1.49 | ***0.017*** |
| 20-Sep | 21.8 ±0.63 | 31.1 ±1.68 | ***0.004*** |
| 21-Sep | 23.5 ±2.75 | 34.4 ±1.82 | ***0.029*** |
| 22-Sep | 28.3 ±0.80 | 37.4 ±2.11 | ***0.013*** |
| 23-Sep | 32.2 ±0.72 | 38.5 ±1.39 | ***0.013*** |
| 24-Sep | 31.3 ±0.27 | 35.7 ±1.29 | ***0.028*** |
| 25-Sep | 28.0 ±0.16 | 31.0 ±0.60 | ***0.006*** |
| 26-Sep | 26.3 ±0.70 | 33.9 ±2.10 | ***0.024*** |
| 27-Sep | 27.2 ±0.85 | 34.9 ±2.25 | ***0.033*** |
| 28-Sep | 24.1 ±0.52 | 26.4 ±2.12 | *0.396* |
| 29-Sep | 26.0 ±0.32 | 32.2 ±1.42 | ***0.010*** |
| 30-Sep | 23.7 ±0.44 | 24.0 ±1.52 | *0.865* |
| 1-Oct | 25.1 ±0.70 | 32.7 ±2.60 | ***0.050*** |
| 2-Oct | 19.8 ±0.92 | 20.6 ±2.33 | *0.791* |
| 3-Oct | 25.9 ±0.36 | 31.9 ±1.83 | ***0.031*** |
| 4-Oct | 28.4 ±0.45 | 33.6 ±1.27 | ***0.016*** |
| 5-Oct | 29.3 ±0.52 | 34.1 ±2.09 | *0.103* |
| 6-Oct | 25.8 ±0.71 | 28.5 ±0.41 | ***0.029*** |
| 7-Oct | 22.9 ±0.73 | 25.9 ±2.81 | *0.406* |
| 8-Oct | 20.3 ±0.12 | 19.7 ±0.81 | *0.564* |
| 9-Oct | 28.2 ±0.60 | 31.4 ±2.45 | *0.315* |
| 10-Oct | 26.5 ±0.43 | 29.7 ±2.77 | *0.361* |
| 11-Oct | 30.9 ±0.66 | 35.2 ±2.58 | *0.211* |
| 12-Oct | 28.2 ±0.39 | 30.7 ±2.28 | *0.385* |
| 13-Oct | 28.1 ±0.60 | 33.1 ±2.79 | *0.183* |
| 14-Oct | 23.4 ±0.27 | 24.2 ±1.61 | *0.686* |
| 15-Oct | 25.4 ±0.15 | 28.9 ±1.85 | *0.149* |
| 16-Oct | 25.8 ±0.51 | 29.6 ±2.70 | *0.279* |
| 17-Oct | 27.5 ±0.47 | 31.7 ±2.32 | *0.173* |
| 18-Oct | 28.7 ±0.68 | 33.9 ±2.41 | *0.121* |
| 19-Oct | 30.1 ±0.72 | 35.2 ±2.51 | *0.145* |
| 20-Oct | 22.0 ±0.59 | 18.3 ±0.83 | ***0.021*** |
| 21-Oct | 21.3 ±0.75 | 23.0 ±2.85 | *0.631* |

| Daytime Mean Temperature (⁰C) | | |
| --- | --- | --- |
| *All farms* | | ***p*-Value** |
| Stable | **Paddock** |  |
| 16.8 ±0.20 | 21.0 ±0.38 | ***0.000*** |
| 15.2 ±0.58 | 18.7 ±0.37 | ***0.005*** |
| 16.6 ±0.43 | 18.9 ±0.39 | ***0.015*** |
| 15.9 ±0.36 | 18.2 ±0.80 | *0.069* |
| 15.0 ±0.22 | 16.7 ±0.71 | *0.091* |
| 14.3 ±0.43 | 17.9 ±0.84 | ***0.016*** |
| 14.4 ±0.75 | 15.9 ±0.89 | *0.305* |
| 17.1 ±0.88 | 18.6 ±0.99 | *0.360* |
| 21.4 ±1.18 | 22.8 ±0.65 | *0.405* |
| 21.9 ±1.88 | 25.5 ±0.19 | *0.148* |
| 15.4 ±0.59 | 13.1 ±1.23 | *0.183* |
| 17.0 ±0.37 | 17.9 ±0.87 | *0.413* |
| 17.3 ±0.35 | 19.0 ±0.90 | *0.185* |
| 16.3 ±0.51 | 18.3 ±1.10 | *0.205* |
| 19.4 ±1.16 | 19.8 ±1.18 | *0.830* |
| 20.1 ±1.15 | 24.1 ±0.50 | ***0.032*** |
| 16.4 ±0.72 | 18.5 ±1.30 | *0.268* |
| 17.3 ±2.13 | 21.5 ±1.17 | *0.194* |
| 23.3 ±0.86 | 24.8 ±1.02 | *0.372* |
| 26.7 ±1.06 | 26.7 ±0.86 | *0.964* |
| 27.4 ±0.61 | 28.9 ±0.85 | *0.259* |
| 22.6 ±0.67 | 24.3 ±0.34 | *0.094* |
| 20.3 ±0.13 | 21.8 ±1.61 | *0.465* |
| 21.6 ±0.61 | 22.5 ±1.54 | *0.643* |
| 21.6 ±0.29 | 20.0 ±1.22 | *0.315* |
| 21.2 ±0.29 | 22.4 ±1.03 | *0.398* |
| 20.2 ±0.31 | 19.7 ±1.04 | *0.697* |
| 18.3 ±0.74 | 21.3 ±1.53 | *0.178* |
| 18.0 ±1.16 | 15.1 ±1.91 | *0.308* |
| 21.1 ±0.67 | 21.5 ±0.34 | *0.642* |
| 22.7 ±0.43 | 23.3 ±0.67 | *0.572* |
| 23.7 ±0.14 | 23.7 ±0.90 | *0.949* |
| 21.3 ±0.90 | 21.5 ±0.26 | *0.797* |
| 19.4 ±0.24 | 18.7 ±1.31 | *0.664* |
| 19.3 ±1.47 | 15.6 ±0.70 | *0.098* |
| 23.6 ±0.14 | 23.6 ±0.93 | *0.996* |
| 22.7 ±0.62 | 22.1 ±0.89 | *0.623* |
| 24.9 ±0.21 | 24.4 ±1.23 | *0.714* |
| 23.4 ±0.53 | 23.4 ±1.14 | *0.958* |
| 21.5 ±0.21 | 21.7 ±1.19 | *0.866* |
| 21.1 ±0.29 | 18.8 ±1.13 | *0.139* |
| 21.2 ±0.23 | 21.1 ±1.00 | *0.916* |
| 21.4 ±0.48 | 20.4 ±1.27 | *0.534* |
| 22.9 ±0.36 | 22.5 ±1.26 | *0.821* |
| 24.1 ±0.37 | 23.8 ±1.39 | *0.844* |
| 24.0 ±0.50 | 24.5 ±1.23 | *0.744* |
| 19.9 ±0.32 | 17.1 ±0.63 | ***0.013*** |
| 18.2 ±0.44 | 16.4 ±1.45 | *0.346* |

***Supplementary Table 2*** *Daytime maximum and mean temperature humidity index (THI) recorded in paddocks and stables during the 2017 breeding seasons (p ≤ 0.05, mean values ± S.E.M.)*

| 19.4 ±0.52 | 17.7 ±0.96 | *0.234* |
| --- | --- | --- |
| 20.6 ±0.60 | 19.3 ±1.00 | *0.365* |
| 23.1 ±0.91 | 20.8 ±1.57 | *0.305* |
| 25.0 ±0.51 | 24.7 ±0.62 | *0.802* |
| 23.7 ±1.03 | 25.2 ±0.93 | *0.404* |
| 21.0 ±0.52 | 17.8 ±1.31 | *0.091* |
| 24.3 ±0.68 | 22.6 ±1.50 | *0.397* |
| 26.7 ±0.57 | 25.9 ±1.40 | *0.663* |
| 24.2 ±1.89 | 27.4 ±0.83 | *0.309* |
| 19.3 ±0.12 | 17.2 ±1.28 | *0.253* |
| 19.7 ±0.33 | 18.1 ±1.42 | *0.397* |
| 21.6 ±0.86 | 19.6 ±1.48 | *0.376* |
| 24.8 ±1.28 | 23.2 ±1.04 | *0.446* |
| 20.6 ±0.38 | 18.6 ±0.43 | ***0.021*** |
| 20.5 ±0.77 | 18.1 ±0.85 | *0.113* |
| 21.3 ±0.43 | 20.9 ±0.34 | *0.636* |
| 20.0 ±0.10 | 18.7 ±1.13 | *0.401* |
| 19.0 ±0.29 | 17.5 ±1.09 | *0.311* |
| 19.8 ±0.42 | 18.5 ±1.35 | *0.477* |
| 20.5 ±0.41 | 19.6 ±1.32 | *0.632* |
| 20.9 ±0.35 | 19.9 ±1.36 | *0.575* |
| 21.2 ±0.47 | 20.1 ±1.41 | *0.572* |
| 21.3 ±0.29 | 20.6 ±1.00 | *0.631* |
| 22.3 ±0.55 | 20.3 ±1.52 | *0.341* |
| 23.0 ±0.56 | 22.3 ±1.28 | *0.665* |
| 21.8 ±0.93 | 19.3 ±0.54 | *0.141* |
| 23.2 ±0.53 | 22.9 ±0.88 | *0.833* |
| 20.4 ±0.39 | 17.5 ±0.74 | ***0.037*** |
| 21.6 ±0.34 | 19.2 ±1.06 | *0.141* |
| 22.3 ±0.38 | 20.6 ±1.10 | *0.281* |
| 20.9 ±0.81 | 20.3 ±0.32 | *0.617* |

| 22-Oct | 23.9 ±0.46 | 26.7 ±1.69 | *0.215* |
| --- | --- | --- | --- |
| 23-Oct | 24.3 ±0.42 | 27.6 ±2.51 | *0.294* |
| 24-Oct | 27.9 ±0.54 | 30.1 ±2.56 | *0.507* |
| 25-Oct | 30.0 ±0.50 | 32.0 ±1.99 | *0.432* |
| 26-Oct | 31.5 ±0.38 | 33.7 ±1.90 | *0.354* |
| 27-Oct | 24.3 ±0.32 | 25.1 ±1.19 | *0.572* |
| 28-Oct | 27.9 ±0.97 | 29.8 ±2.21 | *0.511* |
| 29-Oct | 31.6 ±0.70 | 33.6 ±2.40 | *0.520* |
| 30-Oct | 34.7 ±0.21 | 35.5 ±1.22 | *0.619* |
| 31-Oct | 24.1 ±0.55 | 25.1 ±2.58 | *0.750* |
| 1-Nov | 25.6 ±0.80 | 27.5 ±2.59 | *0.591* |
| 2-Nov | 27.3 ±0.80 | 29.7 ±2.49 | *0.473* |
| 3-Nov | 30.7 ±0.40 | 33.4 ±1.55 | *0.194* |
| 4-Nov | 23.6 ±0.64 | 23.9 ±0.50 | *0.740* |
| 5-Nov | 22.6 ±0.23 | 22.6 ±0.78 | *0.959* |
| 6-Nov | 28.1 ±0.64 | 29.1 ±0.49 | *0.335* |
| 7-Nov | 25.3 ±0.93 | 27.6 ±2.54 | *0.496* |
| 8-Nov | 22.8 ±0.37 | 23.7 ±2.43 | *0.771* |
| 9-Nov | 25.2 ±0.73 | 27.8 ±2.34 | *0.412* |
| 10-Nov | 26.0 ±0.75 | 28.0 ±1.73 | *0.401* |
| 11-Nov | 26.3 ±0.84 | 28.5 ±2.09 | *0.455* |
| 12-Nov | 27.0 ±0.98 | 29.1 ±2.72 | *0.564* |
| 13-Nov | 26.3 ±0.81 | 28.8 ±2.19 | *0.420* |
| 14-Nov | 27.4 ±0.68 | 28.7 ±2.26 | *0.666* |
| 15-Nov | 30.1 ±1.17 | 31.7 ±2.26 | *0.609* |
| 16-Nov | 23.9 ±0.63 | 23.7 ±0.37 | *0.828* |
| 17-Nov | 28.6 ±0.58 | 29.9 ±1.69 | *0.551* |
| 18-Nov | 22.6 ±0.30 | 21.2 ±0.69 | *0.164* |
| 19-Nov | 24.0 ±0.31 | 24.2 ±0.96 | *0.894* |
| 20-Nov | 27.1 ±0.52 | 28.1 ±2.26 | *0.753* |
| 21-Nov | 26.1 ±0.49 | 26.6 ±0.73 | *0.649* |

| Daytime Maximum THI (%) | | | |
| --- | --- | --- | --- |
| Date | ***All farms*** | | ***p*-Value** |
|  | **Stable** | **Paddock** |  |
| 4-Sep | 64.5 ±0.48 | 73.6 ±0.97 | ***0.000*** |
| 5-Sep | 62.8 ±0.50 | 70.2 ±0.57 | ***0.000*** |
| 6-Sep | 63.4 ±0.51 | 70.9 ±0.77 | ***0.000*** |
| 7-Sep | 64.4 ±0.56 | 71.6 ±1.68 | ***0.012*** |
| 8-Sep | 63.8 ±0.67 | 70.7 ±1.58 | ***0.013*** |
| 9-Sep | 63.1 ±0.82 | 74.2 ±2.25 | ***0.007*** |
| 10-Sep | 63.4 ±0.83 | 74.7 ±1.84 | ***0.003*** |
| 11-Sep | 67.3 ±0.50 | 76.6 ±1.52 | ***0.002*** |
| 12-Sep | 71.3 ±0.60 | 78.5 ±1.64 | ***0.005*** |
| 13-Sep | 73.6 ±0.56 | 77.1 ±1.24 | *0.067* |
| 14-Sep | 61.1 ±0.41 | 64.4 ±1.99 | *0.204* |
| 15-Sep | 65.6 ±0.44 | 70.7 ±1.59 | ***0.039*** |
| 16-Sep | 67.5 ±0.38 | 72.5 ±1.73 | ***0.049*** |
| 17-Sep | 65.7 ±0.37 | 74.4 ±1.53 | ***0.004*** |
| 18-Sep | 69.5 ±0.61 | 76.8 ±1.50 | ***0.008*** |
| 19-Sep | 69.9 ±0.29 | 74.6 ±1.47 | ***0.035*** |
| 20-Sep | 65.9 ±0.52 | 74.6 ±1.61 | ***0.004*** |

| Daytime Mean THI (%) | | |
| --- | --- | --- |
| *All farms* | | ***p*-Value** |
| Stable | **Paddock** |  |
| 60.9 ±0.54 | 64.4 ±0.47 | ***0.005*** |
| 58.6 ±0.68 | 62.1 ±0.49 | ***0.011*** |
| 60.4 ±0.53 | 62.4 ±0.44 | ***0.045*** |
| 59.5 ±0.52 | 60.8 ±0.97 | *0.320* |
| 58.6 ±0.53 | 59.4 ±0.89 | *0.530* |
| 57.5 ±0.53 | 60.2 ±0.99 | *0.081* |
| 56.6 ±0.47 | 58.1 ±1.04 | *0.297* |
| 59.1 ±0.26 | 60.5 ±1.13 | *0.329* |
| 63.7 ±0.43 | 65.3 ±0.67 | *0.128* |
| 68.0 ±0.95 | 68.7 ±0.24 | *0.580* |
| 58.6 ±0.41 | 55.2 ±1.62 | *0.133* |
| 60.5 ±0.36 | 61.1 ±0.92 | *0.619* |
| 61.5 ±0.41 | 61.8 ±1.03 | *0.852* |
| 59.3 ±0.28 | 60.9 ±1.26 | *0.347* |
| 61.5 ±0.73 | 61.9 ±1.41 | *0.834* |
| 65.8 ±0.89 | 67.8 ±0.34 | *0.117* |
| 59.1 ±0.34 | 60.9 ±1.46 | *0.332* |

| 59.6 ±2.19 | 63.7 ±1.29 | *0.207* |
| --- | --- | --- |
| 65.9 ±0.84 | 67.4 ±0.95 | *0.347* |
| 69.2 ±0.70 | 69.5 ±0.85 | *0.802* |
| 72.3 ±0.33 | 72.0 ±0.83 | *0.812* |
| 67.5 ±0.85 | 67.6 ±0.33 | *0.889* |
| 64.3 ±0.21 | 64.9 ±1.45 | *0.733* |
| 66.4 ±0.66 | 66.3 ±1.62 | *0.956* |
| 67.3 ±0.21 | 64.7 ±1.34 | *0.156* |
| 65.7 ±0.67 | 65.7 ±1.05 | *0.963* |
| 64.5 ±0.42 | 63.2 ±1.00 | *0.356* |
| 62.6 ±0.42 | 64.1 ±1.52 | *0.442* |
| 60.9 ±0.73 | 58.2 ±2.17 | *0.350* |
| 66.4 ±0.57 | 66.3 ±0.42 | *0.893* |
| 68.3 ±0.63 | 67.7 ±0.67 | *0.590* |
| 70.2 ±0.44 | 68.9 ±0.86 | *0.279* |
| 67.0 ±1.29 | 65.5 ±0.32 | *0.361* |
| 65.0 ±0.22 | 62.9 ±1.34 | *0.251* |
| 63.2 ±0.85 | 59.3 ±0.99 | ***0.042*** |
| 71.6 ±0.24 | 70.0 ±1.11 | *0.267* |
| 69.4 ±0.59 | 67.9 ±0.97 | *0.295* |
| 72.0 ±0.27 | 70.0 ±1.46 | *0.284* |
| 71.1 ±0.77 | 69.4 ±1.23 | *0.350* |
| 66.9 ±0.71 | 65.6 ±1.01 | *0.385* |
| 67.5 ±0.25 | 64.0 ±1.45 | *0.082* |
| 67.3 ±0.40 | 66.0 ±1.36 | *0.452* |
| 66.1 ±0.17 | 64.3 ±1.43 | *0.330* |
| 68.4 ±0.20 | 67.0 ±1.42 | *0.435* |
| 69.9 ±0.19 | 68.4 ±1.61 | *0.464* |
| 70.5 ±0.37 | 68.9 ±1.32 | *0.353* |
| 67.8 ±0.75 | 62.2 ±1.04 | ***0.010*** |
| 62.9 ±0.51 | 60.1 ±1.76 | *0.232* |
| 63.6 ±0.42 | 61.1 ±1.21 | *0.142* |
| 65.6 ±0.42 | 63.6 ±1.14 | *0.222* |
| 67.4 ±0.54 | 64.8 ±1.56 | *0.223* |
| 70.3 ±0.57 | 68.9 ±0.56 | *0.202* |
| 71.7 ±0.54 | 70.4 ±0.95 | *0.349* |
| 66.8 ±0.36 | 62.2 ±1.71 | *0.075* |
| 69.9 ±0.29 | 67.7 ±1.72 | *0.327* |
| 72.8 ±0.30 | 71.3 ±1.44 | *0.418* |
| 71.7 ±0.62 | 71.2 ±0.85 | *0.706* |
| 63.5 ±0.34 | 60.6 ±1.33 | *0.158* |
| 63.6 ±0.25 | 61.3 ±1.56 | *0.291* |
| 65.1 ±0.39 | 62.9 ±1.58 | *0.315* |
| 70.0 ±10.7 | 66.8 ±1.21 | *0.138* |
| 67.6 ±0.61 | 63.9 ±0.73 | ***0.014*** |
| 66.1 ±0.36 | 63.0 ±1.22 | *0.081* |
| 68.5 ±0.61 | 66.4 ±0.76 | *0.106* |
| 64.9 ±0.50 | 62.3 ±1.26 | *0.175* |
| 64.2 ±0.52 | 61.5 ±1.37 | *0.193* |
| 64.2 ±0.28 | 61.9 ±1.63 | *0.308* |
| 65.4 ±0.27 | 63.3 ±1.54 | *0.339* |
| 65.8 ±0.21 | 63.6 ±1.63 | *0.335* |
| 66.2 ±0.17 | 63.9 ±1.52 | *0.286* |

| 21-Sep | 67.1 ±2.76 | 77.0 ±1.49 | *0.034* |
| --- | --- | --- | --- |
| 22-Sep | 71.9 ±0.73 | 79.5 ±1.64 | ***0.011*** |
| 23-Sep | 75.5 ±0.73 | 80.0 ±1.25 | ***0.035*** |
| 24-Sep | 75.1 ±0.27 | 77.9 ±1.11 | *0.080* |
| 25-Sep | 71.5 ±0.23 | 74.3 ±0.59 | ***0.008*** |
| 26-Sep | 70.0 ±0.65 | 76.3 ±1.63 | ***0.021*** |
| 27-Sep | 73.0 ±0.55 | 78.8 ±1.70 | ***0.032*** |
| 28-Sep | 69.7 ±0.37 | 70.9 ±1.60 | *0.534* |
| 29-Sep | 70.5 ±0.24 | 75.5 ±1.29 | ***0.017*** |
| 30-Sep | 67.3 ±0.42 | 67.5 ±1.48 | *0.930* |
| 1-Oct | 68.6 ±0.67 | 75.5 ±2.15 | ***0.038*** |
| 2-Oct | 65.0 ±0.90 | 65.1 ±2.27 | *0.988* |
| 3-Oct | 72.5 ±0.35 | 77.0 ±1.64 | *0.056* |
| 4-Oct | 74.4 ±0.27 | 77.9 ±1.19 | ***0.050*** |
| 5-Oct | 75.3 ±0.37 | 78.8 ±1.82 | *0.157* |
| 6-Oct | 70.7 ±0.94 | 72.9 ±0.48 | *0.119* |
| 7-Oct | 68.6 ±0.51 | 70.8 ±2.52 | *0.501* |
| 8-Oct | 66.6 ±0.26 | 65.2 ±1.01 | *0.297* |
| 9-Oct | 76.8 ±0.22 | 78.0 ±2.26 | *0.645* |
| 10-Oct | 74.5 ±0.25 | 76.1 ±2.46 | *0.594* |
| 11-Oct | 77.3 ±0.53 | 79.7 ±2.28 | *0.413* |
| 12-Oct | 75.7 ±0.40 | 76.1 ±1.68 | *0.843* |
| 13-Oct | 73.5 ±0.21 | 76.7 ±2.22 | *0.263* |
| 14-Oct | 70.5 ±0.17 | 70.5 ±1.80 | *0.988* |
| 15-Oct | 71.6 ±0.30 | 74.6 ±1.70 | *0.177* |
| 16-Oct | 71.4 ±0.40 | 74.1 ±2.44 | *0.382* |
| 17-Oct | 73.5 ±0.27 | 76.2 ±2.11 | *0.307* |
| 18-Oct | 74.9 ±0.55 | 78.1 ±2.38 | *0.291* |
| 19-Oct | 75.9 ±0.63 | 78.8 ±2.21 | *0.310* |
| 20-Oct | 69.4 ±0.68 | 64.2 ±1.34 | ***0.024*** |
| 21-Oct | 66.7 ±0.64 | 67.8 ±2.70 | *0.739* |
| 22-Oct | 69.3 ±0.38 | 71.1 ±1.61 | *0.369* |
| 23-Oct | 70.3 ±0.32 | 72.4 ±2.38 | *0.484* |
| 24-Oct | 73.4 ±0.27 | 74.9 ±1.82 | *0.507* |
| 25-Oct | 74.9 ±0.34 | 75.6 ±1.65 | *0.722* |
| 26-Oct | 77.2 ±0.38 | 78.2 ±1.63 | *0.635* |
| 27-Oct | 71.7 ±0.49 | 71.6 ±1.20 | *0.939* |
| 28-Oct | 74.6 ±0.62 | 75.3 ±2.02 | *0.765* |
| 29-Oct | 78.1 ±0.38 | 78.5 ±2.03 | *0.856* |
| 30-Oct | 78.2 ±0.24 | 78.0 ±1.14 | *0.873* |
| 31-Oct | 68.2 ±0.27 | 68.7 ±2.51 | *0.874* |
| 1-Nov | 69.7 ±0.59 | 71.3 ±2.49 | *0.625* |
| 2-Nov | 72.2 ±0.46 | 73.8 ±2.25 | *0.583* |
| 3-Nov | 74.8 ±0.39 | 76.7 ±1.43 | *0.308* |
| 4-Nov | 71.0 ±0.79 | 70.9 ±0.92 | *0.945* |
| 5-Nov | 69.7 ±0.39 | 69.0 ±1.00 | *0.597* |
| 6-Nov | 75.7 ±0.35 | 75.5 ±0.92 | *0.835* |
| 7-Nov | 70.7 ±0.57 | 72.1 ±2.45 | *0.677* |
| 8-Nov | 68.5 ±0.23 | 68.6 ±2.47 | *0.989* |
| 9-Nov | 70.8 ±0.71 | 72.4 ±2.15 | *0.595* |
| 10-Nov | 71.7 ±0.47 | 72.6 ±1.47 | *0.630* |
| 11-Nov | 72.0 ±0.72 | 72.9 ±1.87 | *0.720* |
| 12-Nov | 72.4 ±0.45 | 73.4 ±2.30 | *0.732* |

***Supplementary Table 3*** *Night-time maximum and mean temperature recorded in paddocks and stables during the 2017 breeding seasons (p ≤ 0.05, mean values ± S.E.M.)*

| 66.2 ±0.19 | 63.9 ±1.20 | *0.362* |
| --- | --- | --- |
| 66.7 ±0.28 | 65.3 ±1.62 | *0.235* |
| 67.4 ±0.08 | 64.6 ±1.43 | *0.261* |
| 68.9 ±0.70 | 66.6 ±0.86 | *0.057* |
| 67.7 ±0.25 | 64.4 ±1.18 | *0.215* |
| 71.1 ±0.71 | 68.9 ±1.19 | ***0.032*** |
| 67.5 ±0.13 | 62.6 ±1.48 | *0.141* |
| 67.7 ±0.49 | 64.3 ±1.27 | *0.171* |
| 68.2 ±1.31 | 65.5 ±0.52 | *0.341* |

| 13-Nov | 72.7 ±0.70 | 73.6 ±2.01 | *0.723* |
| --- | --- | --- | --- |
| 14-Nov | 73.3 ±0.42 | 73.9 ±1.90 | *0.808* |
| 15-Nov | 75.4 ±0.78 | 76.0 ±1.99 | *0.819* |
| 16-Nov | 71.1 ±0.77 | 69.9 ±0.62 | *0.387* |
| 17-Nov | 76.1 ±0.55 | 76.1 ±1.69 | *0.987* |
| 18-Nov | 70.0 ±0.36 | 67.3 ±1.11 | *0.125* |
| 19-Nov | 71.1 ±0.22 | 70.6 ±1.12 | *0.737* |
| 20-Nov | 73.5 ±0.16 | 73.3 ±1.96 | *0.940* |
| 21-Nov | 72.2 ±0.57 | 72.0 ±0.96 | *0.887* |

| Night-time Maximum Temperature (⁰C) | | | |
| --- | --- | --- | --- |
| Date | ***All farms*** | | ***p*-Value** |
|  | **Stable** | **Paddock** |  |
| 4-Sep | 20.0 ±0.44 | 26.7 ±2.07 | ***0.000*** |
| 5-Sep | 18.3 ±0.51 | 22.3 ±1.65 | ***0.000*** |
| 6-Sep | 19.1 ±0.49 | 22.9 ±1.95 | ***0.000*** |
| 7-Sep | 19.8 ±0.49 | 24.5 ±2.67 | ***0.007*** |
| 8-Sep | 19.0 ±0.51 | 23.7 ±2.33 | ***0.009*** |
| 9-Sep | 19.2 ±0.80 | 27.4 ±2.98 | ***0.006*** |
| 10-Sep | 19.1 ±0.71 | 27.8 ±2.95 | ***0.003*** |
| 11-Sep | 23.1 ±0.54 | 29.4 ±2.09 | ***0.002*** |
| 12-Sep | 26.4 ±0.85 | 32.0 ±2.00 | ***0.005*** |
| 13-Sep | 29.2 ±0.49 | 28.6 ±1.74 | *0.068* |
| 14-Sep | 21.0 ±0.99 | 18.2 ±2.02 | *0.179* |
| 15-Sep | 20.6 ±0.38 | 24.1 ±2.10 | ***0.024*** |
| 16-Sep | 22.2 ±0.33 | 24.2 ±1.72 | ***0.031*** |
| 17-Sep | 20.6 ±0.42 | 26.1 ±2.15 | ***0.007*** |
| 18-Sep | 25.0 ±0.73 | 28.0 ±3.38 | ***0.006*** |
| 19-Sep | 24.0 ±0.35 | 27.2 ±1.98 | ***0.017*** |
| 20-Sep | 21.5 ±0.58 | 27.6 ±2.12 | ***0.004*** |
| 21-Sep | 22.6 ±3.44 | 31.2 ±2.25 | ***0.029*** |
| 22-Sep | 28.1 ±0.76 | 32.1 ±3.00 | ***0.013*** |
| 23-Sep | 32.2 ±0.73 | 35.2 ±1.63 | ***0.013*** |
| 24-Sep | 30.0 ±0.39 | 29.1 ±1.96 | ***0.028*** |
| 25-Sep | 26.2 ±0.93 | 25.7 ±0.90 | ***0.006*** |
| 26-Sep | 26.0 ±0.64 | 31.1 ±2.71 | ***0.024*** |
| 27-Sep | 27.2 ±0.76 | 33.2 ±2.09 | ***0.033*** |
| 28-Sep | 23.8 ±0.41 | 22.9 ±2.37 | *0.396* |
| 29-Sep | 25.6 ±0.26 | 29.1 ±1.81 | ***0.010*** |
| 30-Sep | 23.2 ±0.33 | 21.3 ±1.55 | *0.865* |
| 1-Oct | 24.8 ±0.66 | 31.5 ±2.71 | ***0.050*** |
| 2-Oct | 19.6 ±0.67 | 17.5 ±1.30 | *0.791* |
| 3-Oct | 25.4 ±0.38 | 27.5 ±3.21 | ***0.031*** |
| 4-Oct | 28.0 ±0.58 | 30.3 ±2.81 | ***0.016*** |
| 5-Oct | 29.3 ±0.40 | 32.8 ±1.88 | *0.103* |
| 6-Oct | 24.2 ±0.28 | 23.7 ±3.60 | ***0.029*** |
| 7-Oct | 22.3 ±0.65 | 21.6 ±3.17 | *0.406* |
| 8-Oct | 19.7 ±0.29 | 16.4 ±0.85 | *0.564* |
| 9-Oct | 27.3 ±0.90 | 28.5 ±3.26 | *0.315* |
| 10-Oct | 26.0 ±0.51 | 28.6 ±2.93 | *0.361* |
| 11-Oct | 30.9 ±0.72 | 35.1 ±2.87 | *0.211* |
| 12-Oct | 27.8 ±0.48 | 29.7 ±2.54 | *0.385* |
| 13-Oct | 28.0 ±0.68 | 32.9 ±2.72 | *0.183* |
| 14-Oct | 21.2 ±0.27 | 18.3 ±1.03 | *0.686* |
| 15-Oct | 25.1 ±0.31 | 26.5 ±1.80 | *0.149* |

| Night-time Mean Temperature (⁰C) | | |
| --- | --- | --- |
| *All farms* | | ***p*-Value** |
| Stable | **Paddock** |  |
| 15.1 ±1.02 | 13.4 ±0.64 | *0.283* |
| 13.3 ±1.08 | 11.8 ±0.42 | *0.317* |
| 14.3 ±0.65 | 11.0 ±0.70 | ***0.023*** |
| 12.6 ±1.08 | 7.7 ±1.25 | ***0.042*** |
| 11.4 ±1.29 | 7.1 ±0.97 | *0.059* |
| 11.1 ±1.23 | 6.3 ±1.09 | ***0.044*** |
| 10.0 ±1.25 | 4.1 ±1.14 | ***0.023*** |
| 11.0 ±1.71 | 4.5 ±1.26 | ***0.038*** |
| 14.7 ±1.37 | 9.9 ±1.17 | *0.060* |
| 19.1 ±1.16 | 15.2 ±0.34 | ***0.032*** |
| 14.7 ±0.94 | 9.4 ±0.70 | ***0.008*** |
| 12.8 ±1.28 | 7.2 ±0.86 | ***0.020*** |
| 13.8 ±1.07 | 8.2 ±0.92 | ***0.014*** |
| 12.5 ±1.33 | 6.3 ±0.88 | ***0.016*** |
| 12.6 ±1.86 | 8.0 ±0.76 | *0.092* |
| 15.9 ±1.53 | 11.2 ±0.89 | *0.062* |
| 11.6 ±1.31 | 5.6 ±1.34 | ***0.033*** |
| 10.9 ±1.64 | 8.0 ±1.20 | *0.253* |
| 16.4 ±2.04 | 10.6 ±1.17 | *0.075* |
| 20.4 ±1.67 | 18.3 ±0.76 | *0.352* |
| 24.7 ±1.20 | 21.8 ±0.46 | *0.103* |
| 19.7 ±1.42 | 14.7 ±0.59 | ***0.030*** |
| 16.1 ±1.41 | 10.9 ±1.03 | ***0.042*** |
| 17.7 ±0.96 | 13.7 ±1037 | *0.087* |
| 19.6 ±0.54 | 15.3 ±0.76 | ***0.006*** |
| 17.1 ±1.43 | 12.8 ±0.63 | *0.055* |
| 17.7 ±1.19 | 13.6 ±0.50 | ***0.035*** |
| 14.3 ±1.14 | 9.3 ±1.22 | ***0.042*** |
| 13.3 ±1.32 | 9.2 ±1.05 | *0.080* |
| 18.4 ±0.64 | 15.0 ±0.90 | ***0.037*** |
| 18.8 ±1.21 | 15.1 ±1.06 | *0.092* |
| 20.7 ±0.79 | 17.1 ±1.19 | *0.070* |
| 19.3 ±0.78 | 14.7 ±0.81 | ***0.012*** |
| 18.0 ±0.33 | 13.5 ±1.40 | ***0.036*** |
| 16.4 ±0.66 | 11.7 ±1.09 | ***0.018*** |
| 20.6 ±0.54 | 17.2 ±1.31 | *0.084* |
| 20.4 ±0.43 | 15.9 ±1.23 | ***0.024*** |
| 22.3 ±1.07 | 19.4 ±1.37 | *0.210* |
| 21.4 ±0.88 | 17.7 ±1.33 | *0.087* |
| 18.0 ±1.14 | 14.2 ±1.11 | *0.083* |
| 19.0 ±0.40 | 14.6 ±1.03 | ***0.014*** |
| 18.8 ±0.64 | 14.8 ±1.51 | *0.080* |

***Supplementary Table 4*** *Night-time maximum and mean temperature humidity index (THI) recorded in paddocks and stables during the 2017 breeding seasons (p ≤ 0.05, mean values ± S.E.M.)*

| 17.1 ±1.23 | 12.9 ±1.74 | *0.142* |
| --- | --- | --- |
| 19.1 ±0.93 | 15.3 ±1.65 | *0.129* |
| 20.6 ±1.09 | 17.6 ±1.49 | *0.211* |
| 21.4 ±1.31 | 18.6 ±1.17 | *0.214* |
| 19.4 ±0.61 | 14.6 ±0.85 | ***0.008*** |
| 16.7 ±0.74 | 12.5 ±1.37 | *0.059* |
| 15.7 ±1.41 | 11.6 ±1.29 | *0.112* |
| 17.9 ±0.73 | 13.9 ±1.45 | *0.081* |
| 18.2 ±1.36 | 14.7 ±1.24 | *0.152* |
| 20.4 ±0.35 | 15.4 ±1.17 | ***0.012*** |
| 21.3 ±0.65 | 16.9 ±1.38 | ***0.044*** |
| 19.8 ±0.58 | 15.8 ±1.39 | *0.060* |
| 20.6 ±0.76 | 16.8 ±1.19 | *0.060* |
| 23.9 ±0.70 | 21.0 ±1.03 | *0.086* |
| 21.9 ±1.04 | 18.2 ±0.75 | *0.077* |
| 18.3 ±0.78 | 14.0 ±1.21 | *0.054* |
| 17.4 ±1.25 | 13.5 ±1.07 | *0.114* |
| 17.3 ±1.97 | 14.5 ±1.24 | *0.386* |
| 21.4 ±1.99 | 17.0 ±1.02 | *0.145* |
| 19.9 ±0.47 | 15.6 ±0.81 | ***0.007*** |
| 18.2 ±0.53 | 14.2 ±0.93 | ***0.017*** |
| 18.8 ±0.71 | 14.9 ±1.15 | ***0.047*** |
| 16.7 ±1.52 | 13.4 ±0.97 | *0.220* |
| 18.2 ±0.62 | 14.2 ±1.24 | *0.062* |
| 16.2 ±1.96 | 12.8 ±1.53 | *0.325* |
| 16.9 ±1.89 | 13.4 ±1.36 | *0.285* |
| 17.4 ±1.70 | 14.0 ±1.48 | *0.273* |
| 17.9 ±1.57 | 14.5 ±1.52 | *0.257* |
| 18.1 ±1.51 | 15.1 ±1.30 | *0.271* |
| 18.9 ±1.90 | 16.1 ±1.55 | *0.383* |
| 19.7 ±1.78 | 16.9 ±1.22 | *0.347* |
| 19.5 ±1.01 | 16.0 ±0.84 | *0.090* |
| 20.9 ±0.96 | 17.7 ±1.02 | *0.114* |
| 20.4 ±0.53 | 16.2 ±1.18 | ***0.047*** |
| 19.1 ±0.74 | 15.6 ±1.08 | *0.074* |
| 19.7 ±1.00 | 16.9 ±1.17 | *0.188* |
| 19.5 ±1.41 | 15.6 ±0.43 | *0.285* |

| 16-Oct | 25.3 ±0.60 | 28.0 ±2.83 | *0.279* |
| --- | --- | --- | --- |
| 17-Oct | 27.0 ±0.60 | 30.9 ±2.56 | *0.173* |
| 18-Oct | 28.4 ±0.64 | 32.9 ±1.99 | *0.121* |
| 19-Oct | 30.0 ±0.78 | 36.0 ±2.40 | *0.145* |
| 20-Oct | 22.9 ±0.76 | 18.5 ±0.62 | ***0.021*** |
| 21-Oct | 21.2 ±0.65 | 24.0 ±2.20 | *0.631* |
| 22-Oct | 23.2 ±0.60 | 22.9 ±1.45 | *0.215* |
| 23-Oct | 23.7 ±0.63 | 26.1 ±2.35 | *0.294* |
| 24-Oct | 27.8 ±0.69 | 30.5 ±2.75 | *0.507* |
| 25-Oct | 28.2 ±0.48 | 26.9 ±1.61 | *0.432* |
| 26-Oct | 29.7 ±0.59 | 29.8 ±2.35 | *0.354* |
| 27-Oct | 24.9 ±0.64 | 27.9 ±2.41 | *0.572* |
| 28-Oct | 27.3 ±1.01 | 26.6 ±2.30 | *0.511* |
| 29-Oct | 31.5 ±0.66 | 33.3 ±2.02 | *0.520* |
| 30-Oct | 33.5 ±0.64 | 33.5 ±1.02 | *0.619* |
| 31-Oct | 24.1 ±0.69 | 25.6 ±2.79 | *0.750* |
| 1-Nov | 25.9 ±0.72 | 27.8 ±2.45 | *0.591* |
| 2-Nov | 27.4 ±0.86 | 31.2 ±2.56 | *0.473* |
| 3-Nov | 30.3 ±0.31 | 32.0 ±1.65 | *0.194* |
| 4-Nov | 23.2 ±0.26 | 20.2 ±0.84 | *0.740* |
| 5-Nov | 22.2 ±0.34 | 22.0 ±1.31 | *0.959* |
| 6-Nov | 24.4 ±0.41 | 25.0 ±1.93 | *0.335* |
| 7-Nov | 24.9 ±0.61 | 27.4 ±2.22 | *0.496* |
| 8-Nov | 22.5 ±0.45 | 23.8 ±2.35 | *0.771* |
| 9-Nov | 25.1 ±0.66 | 26.9 ±2.18 | *0.412* |
| 10-Nov | 25.5 ±0.50 | 26.3 ±1.37 | *0.401* |
| 11-Nov | 26.2 ±0.83 | 27.5 ±2.26 | *0.455* |
| 12-Nov | 26.8 ±0.82 | 28.8 ±2.52 | *0.564* |
| 13-Nov | 26.1 ±0.81 | 28.0 ±2.18 | *0.420* |
| 14-Nov | 27.2 ±0.54 | 29.1 ±2.30 | *0.666* |
| 15-Nov | 29.0 ±0.42 | 30.1 ±1.52 | *0.609* |
| 16-Nov | 23.7 ±0.61 | 21.9 ±0.66 | *0.828* |
| 17-Nov | 27.9 ±0.75 | 25.7 ±2.53 | *0.551* |
| 18-Nov | 23.2 ±0.48 | 19.3 ±1.09 | *0.164* |
| 19-Nov | 23.9 ±0.33 | 22.8 ±1.98 | *0.894* |
| 20-Nov | 27.0 ±0.61 | 27.4 ±2.33 | *0.753* |
| 21-Nov | 26.5 ±0.14 | 23.5 ±1.69 | *0.649* |

| Night-time Maximum THI (%) | | | |
| --- | --- | --- | --- |
| Date | ***All farms*** | | ***p*-Value** |
|  | **Stable** | **Paddock** |  |
| 4-Sep | 63.9 ±0.40 | 70.2 ±1.98 | ***0.036*** |
| 5-Sep | 62.1 ±0.57 | 65.8 ±1.61 | *0.108* |
| 6-Sep | 63.0 ±0.56 | 66.3 ±1.62 | *0.193* |
| 7-Sep | 63.8 ±0.52 | 68.1 ±2.55 | *0.204* |
| 8-Sep | 63.1 ±0.59 | 67.3 ±2.24 | *0.170* |
| 9-Sep | 63.1 ±0.76 | 70.7 ±2.95 | *0.071* |
| 10-Sep | 63.3 ±0.65 | 71.1 ±2.79 | *0.056* |
| 11-Sep | 67.0 ±0.42 | 72.7 ±2.07 | *0.058* |
| 12-Sep | 70.6 ±0.73 | 75.3 ±1.86 | *0.087* |
| 13-Sep | 72.6 ±0.48 | 72.0 ±1.72 | *0.780* |
| 14-Sep | 65.9 ±1.15 | 61.7 ±2.01 | *0.163* |

| Night-time Mean THI (%) | | |
| --- | --- | --- |
| *All farms* | | ***p*-Value** |
| Stable | **Paddock** |  |
| 58.5 ±1.24 | 56.3 ±0.84 | *0.262* |
| 56.4 ±1.36 | 54.5 ±0.65 | *0.335* |
| 57.7 ±0.80 | 53.4 ±0.88 | ***0.020*** |
| 55.3 ±1.44 | 48.1 ±1.88 | ***0.040*** |
| 53.6 ±1.79 | 47.0 ±1.49 | ***0.049*** |
| 53.4 ±1.69 | 46.1 ±1.57 | ***0.034*** |
| 51.7 ±1.81 | 41.9 ±1.90 | ***0.018*** |
| 52.7 ±2.35 | 42.5 ±1.90 | ***0.027*** |
| 57.6 ±1.92 | 50.5 ±1.84 | *0.059* |
| 62.9 ±1.50 | 57.9 ±0.59 | ***0.037*** |
| 58.2 ±1.20 | 51.0 ±0.99 | ***0.007*** |

| 55.5 ±1.83 | 51.0 ±1.40 | *0.019* |
| --- | --- | --- |
| 56.7 ±1.50 | 47.9 ±1.55 | ***0.012*** |
| 55.0 ±1.90 | 44.8 ±1.49 | ***0.011*** |
| 54.8 ±2.59 | 47.0 ±1.27 | *0.057* |
| 59.2 ±2.01 | 53.0 ±1.31 | *0.066* |
| 53.9 ± 1.77 | 44.4 ±2.20 | ***0.027*** |
| 52.6 ±2.16 | 47.4 ±1.91 | *0.174* |
| 59.6 ±2.45 | 51.7 ±1.71 | *0.062* |
| 63.9 ± 1.69 | 60.6 ±0.92 | *0.188* |
| 68.9 ± 1.05 | 65.3 ±0.44 | ***0.037*** |
| 63.7 ±1.65 | 57.5 ±080 | ***0.026*** |
| 59.6 ±1.74 | 52.5 ±1.46 | ***0.034*** |
| 62.0 ±1.24 | 55.6 ±1.97 | *0.056* |
| 64.9 ± 0.87 | 58.7 ±0.97 | ***0.006*** |
| 60.8 ±1.83 | 54.5 ±0.98 | ***0.039*** |
| 61.4 ±1.38 | 56.6 ±063 | ***0.033*** |
| 57.2 ±1.43 | 50.2 ±1.82 | ***0.040*** |
| 56.4 ±1.90 | 50.3 ±1.59 | *0.076* |
| 63.4 ±0.88 | 57.9 ±1.15 | ***0.016*** |
| 63.7 ±1.68 | 57.7 ±1.40 | *0.053* |
| 66.3 ±0.83 | 60.3 ±1.61 | ***0.030*** |
| 64.2 ±1.15 | 57.7 ±1.02 | ***0.011*** |
| 62.9 ± 0.42 | 56.2 ±1.91 | ***0.025*** |
| 61.2 ±1.02 | 53.5 ±1.85 | ***0.019*** |
| 67.2 ±0.66 | 61.4 ±1.80 | ***0.041*** |
| 66.8 ±0.72 | 59.4 ±1.72 | ***0.014*** |
| 68.6 ±1.16 | 63.6 ±1.79 | *0.090* |
| 66.8 ±1.08 | 61.5 ±1.69 | *0.061* |
| 62.3 ±1.54 | 55.9 ±1.41 | ***0.039*** |
| 64.8 ±0.60 | 58.0 ±1.57 | ***0.013*** |
| 64.0 ±0.76 | 57.7 ±2.26 | *0.063* |
| 61.2 ±1.65 | 54.5 ±2.41 | *0.093* |
| 64.1 ±1.15 | 58.0 ±2.24 | *0.080* |
| 65.9 ± 1.25 | 61.0 ±1.95 | *0.115* |
| 66.6 ±1.49 | 62.1 ±1.35 | *0.097* |
| 65.2 ±0.82 | 57.9 ±1.34 | ***0.007*** |
| 61.0 ±1.03 | 54.3 ±1.96 | ***0.040*** |
| 59.3 ±2.00 | 52.5 ±1.98 | *0.082* |
| 62.6 ±0.95 | 56.5 ±1.94 | ***0.050*** |
| 62.3 ±1.71 | 56.5 ±1.68 | *0.082* |
| 66.0 ±0.59 | 58.8 ±1.57 | ***0.010*** |
| 67.2 ±0.72 | 60.3 ±1.62 | ***0.015*** |
| 65.8 ±0.75 | 59.1 ±1.91 | ***0.031*** |
| 66.4 ±0.84 | 60.5 ±1.59 | ***0.028*** |
| 69.7 ±0.58 | 65.4 ±1.20 | ***0.031*** |
| 66.7 ±0.97 | 61.4 ±1.04 | ***0.028*** |
| 62.3 ±0.88 | 56.9 ±1.37 | ***0.038*** |
| 61.2 ±1.47 | 55.1 ±1.44 | *0.062* |
| 61.1 ±2.51 | 56.4 ±1.68 | *0.274* |
| 65.7 ±2.17 | 59.5 ±1.37 | *0.080* |
| 66.2 ±0.71 | 59.6 ±1.21 | ***0.006*** |
| 63.6 ±0.77 | 57.3 ±1.45 | ***0.016*** |
| 64.0 ±0.85 | 58.1 ±1.61 | ***0.031*** |

| 15-Sep | 65.1 ±0.44 | 67.8 ±2.03 | *0.301* |
| --- | --- | --- | --- |
| 16-Sep | 66.6 ±0.44 | 67.8 ±1.68 | *0.566* |
| 17-Sep | 65.2 ±0.34 | 69.9 ±1.99 | *0.090* |
| 18-Sep | 69.3 ±0.58 | 71.7 ±2.94 | *0.518* |
| 19-Sep | 67.4 ±0.37 | 70.6 ±1.96 | *0.224* |
| 20-Sep | 65.6 ±0.48 | 71.0 ±2.09 | *0.072* |
| 21-Sep | 65.9 ±3.69 | 74.5 ±2.23 | *0.135* |
| 22-Sep | 71.7 ±0.69 | 74.5 ±2.49 | *0.387* |
| 23-Sep | 75.4 ±0.75 | 77.6 ±1.27 | *0.245* |
| 24-Sep | 74.0 ±0.58 | 72.4 ±1.91 | *0.523* |
| 25-Sep | 69.6 ±0.94 | 69.1 ±0.89 | *0.771* |
| 26-Sep | 69.8 ±0.60 | 75.4 ±2.16 | *0.072* |
| 27-Sep | 73.1 ±0.45 | 77.3 ±1.48 | *0.057* |
| 28-Sep | 68.8 ±0.35 | 67.1 ±2.11 | *0.524* |
| 29-Sep | 69.8 ±0.38 | 72.5 ±1.77 | *0.238* |
| 30-Sep | 66.7 ±0.31 | 64.7 ±1.53 | *0.321* |
| 1-Oct | 68.3 ±0.64 | 74.3 ±2.52 | *0.092* |
| 2-Oct | 64.9 ±0.58 | 61.7 ±1.38 | *0.113* |
| 3-Oct | 71.7 ±0.37 | 72.3 ±2.84 | *0.880* |
| 4-Oct | 73.9 ±0.45 | 74.7 ±2.58 | *0.817* |
| 5-Oct | 74.9 ±0.42 | 77.0 ±1.64 | *0.334* |
| 6-Oct | 68.7 ±0.58 | 67.5 ±3.46 | *0.791* |
| 7-Oct | 68.0 ±0.56 | 66.1 ±3.10 | *0.614* |
| 8-Oct | 66.3 ±0.47 | 61.0 ±1.29 | ***0.017*** |
| 9-Oct | 75.4 ±0.70 | 75.0 ±2.78 | *0.903* |
| 10-Oct | 73.9 ±0.44 | 74.7 ±2.52 | *0.780* |
| 11-Oct | 77.1 ±0.68 | 79.2 ±2.55 | *0.523* |
| 12-Oct | 73.4 ±0.79 | 73.8 ±2.29 | *0.891* |
| 13-Oct | 73.3 ±0.33 | 76.5 ±1.97 | *0.213* |
| 14-Oct | 67.8 ±0.46 | 63.4 ±1.39 | ***0.041*** |
| 15-Oct | 71.0 ±0.43 | 71.7 ±1.99 | *0.783* |
| 16-Oct | 70.9 ±0.51 | 72.6 ±2.52 | *0.586* |
| 17-Oct | 72.8 ±0.48 | 75.3 ±2.29 | *0.392* |
| 18-Oct | 74.4 ±0.57 | 76.9 ±1.73 | *0.274* |
| 19-Oct | 75.6 ±0.56 | 79.1 ±1.79 | *0.149* |
| 20-Oct | 69.1 ±1.04 | 63.4 ±0.83 | ***0.010*** |
| 21-Oct | 66.4 ±0.57 | 68.8 ±2.08 | *0.381* |
| 22-Oct | 68.5 ±0.32 | 67.6 ±1.53 | *0.652* |
| 23-Oct | 69.7 ±0.55 | 71.1 ±2.10 | *0.596* |
| 24-Oct | 73.1 ±0.45 | 75.3 ±1.81 | *0.356* |
| 25-Oct | 73.5 ±0.57 | 71.2 ±1.57 | *0.286* |
| 26-Oct | 75.1 ±0.51 | 73.7 ±1.97 | *0.560* |
| 27-Oct | 72.3 ±0.56 | 74.2 ±2.23 | *0.503* |
| 28-Oct | 74.1 ±0.62 | 71.9 ±1.87 | *0.369* |
| 29-Oct | 76.9 ±0.88 | 78.0 ±1.68 | *0.631* |
| 30-Oct | 76.6 ±0.72 | 76.5 ±1.08 | *0.926* |
| 31-Oct | 68.3 ±0.46 | 69.0 ±2.73 | *0.839* |
| 1-Nov | 69.9 ±0.59 | 71.4 ±2.37 | *0.621* |
| 2-Nov | 72.2 ±0.49 | 74.6 ±2.11 | *0.398* |
| 3-Nov | 74.4 ±0.35 | 75.1 ±1.68 | *0.748* |
| 4-Nov | 69.6 ±0.51 | 65.6 ±1.00 | ***0.022*** |
| 5-Nov | 69.0 ±0.52 | 68.0 ±1.63 | *0.609* |
| 6-Nov | 71.4 ±0.47 | 71.2 ±2.22 | *0.940* |

| 60.6 ±1.99 | 55.6 ±1.28 | *0.165* |
| --- | --- | --- |
| 63.0 ±0.82 | 57.1 ±1.65 | ***0.046*** |
| 59.9 ±2.64 | 54.2 ±2.17 | *0.239* |
| 60.8 ±2.56 | 55.2 ±1.97 | *0.220* |
| 61.5 ±2.26 | 56.0 ±2.06 | *0.196* |
| 62.3 ±2.09 | 56.7 ±2.06 | *0.177* |
| 62.8 ±2.01 | 57.8 ±1.87 | *0.194* |
| 63.7 ±2.47 | 59.1 ±2.13 | *0.295* |
| 64.5 ±2.10 | 59.9 ±1.71 | *0.222* |
| 65.4 ±1.33 | 60.2 ±1.34 | *0.072* |
| 67.3 ±1.13 | 62.4 ±1.43 | *0.068* |
| 67.2 ±0.69 | 60.5 ±1.82 | ***0.043*** |
| 64.8 ±0.94 | 59.1 ±1.59 | *0.051* |
| 65.3 ±1.28 | 60.8 ±1.46 | *0.109* |
| 63.8 ±1.63 | 59.2 ±0.63 | *0.135* |

| 7-Nov | 70.4 ±0.54 | 71.6 ±2.09 | *0.651* |
| --- | --- | --- | --- |
| 8-Nov | 68.0 ±0.20 | 68.5 ±2.27 | *0.866* |
| 9-Nov | 70.7 ±0.57 | 71.2 ±2.00 | *0.840* |
| 10-Nov | 71.0 ±0.31 | 70.8 ±1.17 | *0.897* |
| 11-Nov | 71.7 ±0.57 | 72.0 ±1.98 | *0.919* |
| 12-Nov | 72.1 ±0.27 | 73.3 ±2.20 | *0.681* |
| 13-Nov | 72.3 ±0.73 | 72.9 ±2.06 | *0.805* |
| 14-Nov | 72.9 ±0.35 | 74.1 ±1.87 | *0.626* |
| 15-Nov | 74.2 ±0.37 | 74.2 ±1.47 | *0.983* |
| 16-Nov | 71.1 ±0.68 | 68.1 ±1.10 | *0.108* |
| 17-Nov | 75.1 ±0.64 | 71.7 ±2.61 | *0.354* |
| 18-Nov | 70.4 ±0.58 | 65.0 ±1.60 | *0.054* |
| 19-Nov | 70.7 ±0.26 | 68.4 ±2.14 | *0.435* |
| 20-Nov | 73.5 ±0.15 | 72.7 ±1.94 | *0.761* |
| 21-Nov | 70.1 ±2.29 | 68.7 ±1.66 | *0.716* |
